# Discovery of a gene cluster for the biosynthesis of novel cyclic peptide compound, KK-1, in *Curvularia clavata*

**DOI:** 10.3389/ffunb.2022.1081179

**Published:** 2023-01-20

**Authors:** Shigenari Yamaguchi, Tomonori Fujioka, Akira Yoshimi, Toshitaka Kumagai, Maiko Umemura, Keietsu Abe, Masayuki Machida, Kiyoshi Kawai

**Affiliations:** ^1^ Biotechnology Laboratory, Life & Environment Research Center, Life Science Research Institute, Research & Development Division, Kumiai Chemical Industry Co., Ltd., Shizuoka, Japan; ^2^ ABE-Project, New Industry Creation Hatchery Center, Tohoku University, Sendai, Japan; ^3^ Laboratory of Terrestrial Microbial Ecology, Graduate School of Agriculture, Kyoto University, Kyoto, Japan; ^4^ Fermlab Inc., Tokyo, Japan; ^5^ Bio-system Research Group, Bioproduction Research Institute, National Institute of Advanced Industrial Science and Technology (AIST), Tsukuba, Japan; ^6^ Laboratory of Applied Microbiology, Graduate School of Agricultural Science, Tohoku University, Sendai, Japan; ^7^ Graduate School of Engineering, Genome Biotechnology Laboratory, Kanazawa Institute of Technology, Ishikawa, Japan

**Keywords:** non-ribosomal peptide, biosynthetic gene cluster, antifungal agent, genome sequence, *Curvularia clavata*, natural fungicide, pesticide, grey mold

## Abstract

KK-1, a cyclic depsipeptide with 10 residues produced by a filamentous fungus *Curvularia clavata* BAUA-2787, is a promising pesticide active compound with high activity against many plant pathogens, especially *Botrytis cinerea*. As a first step toward the future mass production of KK-1 through synthetic biological approaches, we aimed to identify the genes responsible for the KK-1 biosynthesis. To achieve this, we conducted whole genome sequencing and transcriptome analysis of *C. clavata* BAUA-2787 to predict the KK-1 biosynthetic gene cluster. We then generated the overexpression and deletion mutants for each cluster gene using our originally developed transformation system for this fungus, and analyzed the KK-1 production and the cluster gene expression levels to confirm their involvement in KK-1 biosynthesis. As a result of these, a region of approximately 71 kb was found, containing 10 open reading frames, which were co-induced during KK-1 production, as a biosynthetic gene cluster. These include *kk1B*, which encodes nonribosomal peptide synthetase with a domain structure that is consistent with the structural features of KK-1, and *kk1F*, which encodes a transcription factor. The overexpression of *kk1F* increased the expression of the entire cluster genes and, consequently, improved KK-1 production, whereas its deletion decreased the expression of the entire cluster genes and almost eliminated KK-1 production, demonstrating that the protein encoded by *kk1F* regulates the expressions of the other nine cluster genes cooperatively as the pathway-specific transcription factor. Furthermore, the deletion of each cluster gene caused a reduction in KK-1 productivity, indicating that each gene is involved in KK-1 production. The genes *kk1A*, *kk1D*, *kk1H*, and *kk1I*, which showed a significant decrease in KK-1 productivity due to deletion, were presumed to be directly involved in KK-1 structure formation, including the biosynthesis of the constituent residues. *kk1C*, *kk1E*, *kk1G*, and *kk1J*, which maintained a certain level of KK-1 productivity despite deletion, were possibly involved in promoting or assisting KK-1 production, such as extracellular transportation and the removal of aberrant units incorporated into the peptide chain.

## Introduction

With growing global interest in the realization of a sustainable society, the reduction of environmental impacts in food systems is recognized as one of the most important issues worldwide (Brzozowski and Mazourek, 2018; Meemken and Qaim, 2018; Lazaroiu et al., 2019). Particularly in Europe, there has been strong concern about the impact on environment and biodiversity due to excessive dependence on chemical pesticides in agriculture. This has led the European Commission to establish concrete numerical targets to be achieved by 2030 for reducing the use of chemical pesticides and promoting organic agriculture in its “Farm to Fork Strategy,” which was released in May 2020 (https://food.ec.europa.eu/system/files/2020-05/f2f_action-plan_2020_strategy-info_en.pdf). This has been followed by a worldwide movement to tighten regulations on chemical pesticides, such as Japan’s strategy for sustainable food systems, called MeaDRI (https://www.maff.go.jp/e/policies/env/env_policy/meadri.html). Accordingly, the demand for new pesticides of natural origin, which are considered to have a relatively low impact on the environment and living organisms, is expected to increase further in the future.

The secondary metabolites produced by microorganisms such as fungi, actinomycetes, and bacteria, are diverse and include many compounds that exhibit remarkable biological activity (Pham et al., 2019; Jakubczyk and Dussart, 2020; De Simeis and Serra, 2021). Many of them have complex chemical structures based on carbon skeletons that result from characteristic biosynthetic mechanisms, including polyketide synthases, non-ribosomal peptide synthetases and terpene synthases (Pham et al., 2019; Jakubczyk and Dussart, 2020; De Simeis and Serra, 2021). When natural compounds with such complex structures have pesticidal activity, they are expected to act on different targets from existing chemical pesticides, leading to the development of pesticides with less environmental impacts, and providing a solution to important problems in modern agriculture, such as pesticide resistance in pests and weeds.

Some of the secondary metabolites produced by microorganisms have been commercialized as pesticides (Cantrell et al., 2012). For example, polyoxins are nucleoside antibiotics that are isolated from *Streptomyces cacaoi* and are mainly used for the control of plant pathogens (Isono et al., 1965; Isono et al., 1967). Polyoxins are a mixture of structurally similar compounds whose basic structure is composed of three moieties including the nucleoside skeleton, polyoxamic acid, and carbamoylpolyoxamic acid (Isono et al., 1969; Uramoto et al., 1981); and they are the only pesticides that specifically inhibit the biosynthesis of chitin (Endo and Misato, 1969; Endo et al., 1970), a major component of the fungal cell wall. Spinosyns are isolated from *Saccharopolyspora spinosa* and are macrolide compounds with insecticidal activity (Mertz and Yao, 1990; Kirst et al., 1992). The main products in the fermentation cultures are spinosyn A and spinosyn D (Kirst, 2010), and a mixture of these two has been commercialized as Spinosad. The basic structure consists of a tetracycle containing a 12-membered macrocyclic lactone attached to a natural sugar, rhamnose, and an amino sugar, forosamine (Kirst et al., 1992). Spinosyns exhibit insecticidal activity by acting on the nicotinic acetylcholine receptors in the insect neurotransmitter system (Orr et al., 2009; Crossthwaite et al., 2017). In addition to the metabolites that are specified above, many other secondary metabolites that exhibit pesticidal activity have been isolated; however, in many cases commercialization is difficult even if promising activity is confirmed. One of the major causes of this is the cost. When considering the industrial production of secondary metabolites with complex structures, fermentation methods with a microorganism, such as polyoxins and spinosyns, are a realistic production method, since synthetic organic chemistry methods are not profitable. However, in general, most of these secondary metabolites are produced in small quantities and often only under specific conditions. This makes it very difficult to ensure cost-effective and stable production as pesticides, which is a barrier to their practical application. To date, some progress has been made in improving productivity, especially in the field of pharmaceuticals, through the optimization of culture conditions and breeding by introducing random mutations (Parekh et al., 2000; Lee et al., 2002; Korbekandi et al., 2010; Siddique et al., 2014). However, it is difficult to achieve commercialization using only these methods because it is likely to take a long time to obtain high-producing strains, and agrochemicals need to be mass-produced at a lower cost than pharmaceuticals.

One solution to efficiently produce such valuable secondary metabolites is a synthetic biology approach that has recently attracted attention (Katz et al., 2018; Choi et al., 2019). With the recent development of next generation sequencing and bioinformatics, it has become easier to analyze genome information and the gene functions of microorganisms (Zhang et al., 2017; Hu et al., 2021). This has also facilitated the identification of genes that are related to the biosynthesis of certain secondary metabolites and the elucidation of biosynthetic pathways (Zhang et al., 2017). The cloning of these biosynthetic genes and their introduction into appropriate hosts, and the construction of systems that are specialized for the production of target compounds in the host by modifying these genes, i.e., the synthetic biological approach (Siddiqui et al., 2012; Jensen and Keasling, 2015; Lee et al., 2019), is expected to realize a large supply of natural products with useful activities. These approaches for the efficient production of useful compounds have made rapid progress in recent years (Cravens et al., 2019; Yang et al., 2020; Zhang et al., 2021). For example, in the pharmaceutical field, the productivity of the precursor of artemisinin, a terpenoid with antimalarial activity that was extracted from *Artemisia annua*, a kind of herb, was dramatically improved (Westfall et al., 2012; Paddon et al., 2013; Kung et al., 2018). Therefore, these approaches expand the possibilities for the efficient mass production and stable supply of natural compounds with potential agrochemical activity, which have previously been difficult to put into practical use due to their low productivity.

**Figure 1:**
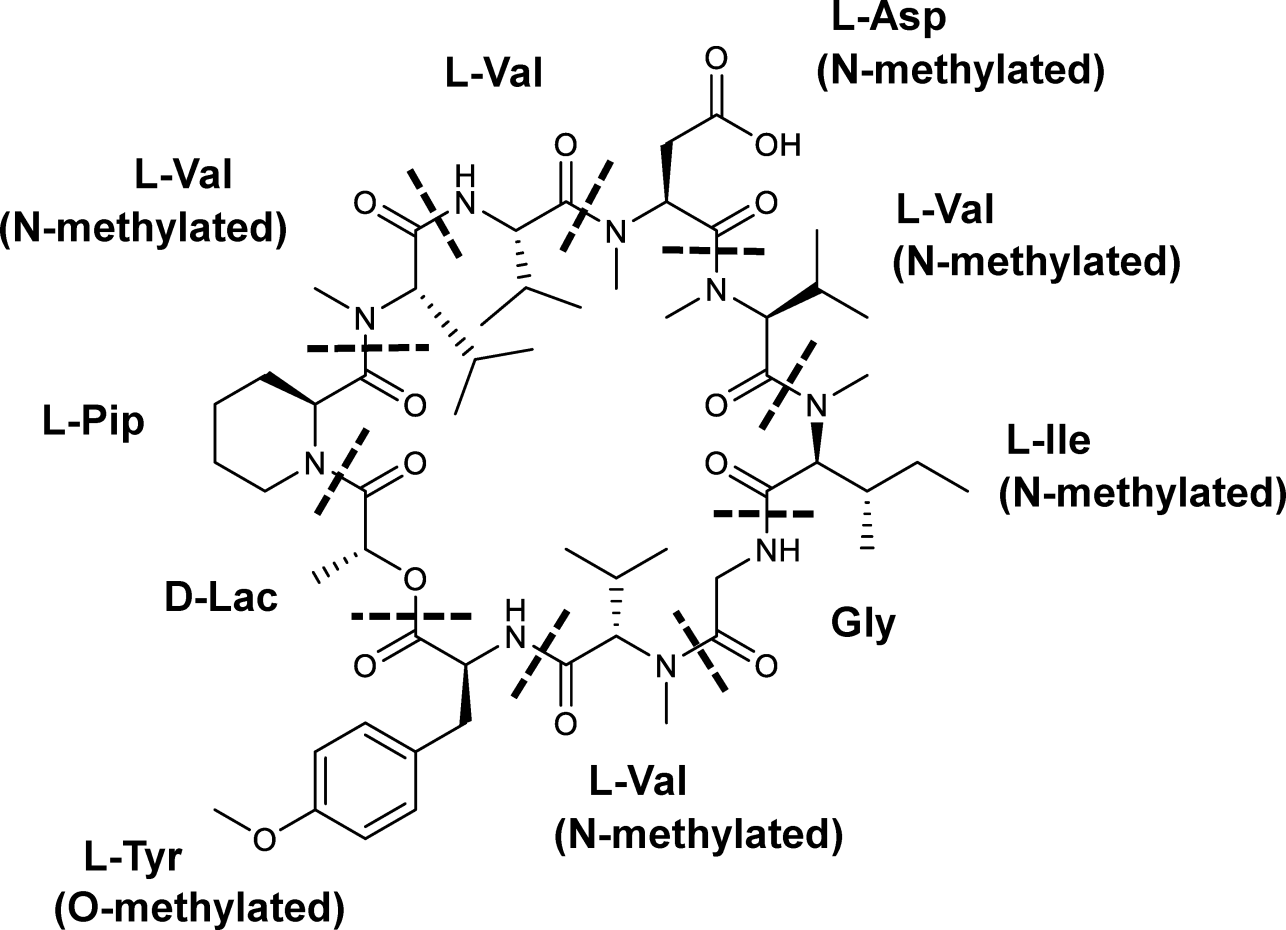
The chemical structure of KK-1. The constituent residues are separated by dotted lines: Lac, lactic acid residue; Pip, pipecolic acid residue; Val, valine residue; Asp, aspartic acid residue; Ile, isoleucine residue; Gly, glycine residue; Tyr, tyrosine residue.

Here, we focused on an antifungal compound (CAS No. 143380-71-6), hereinafter called KK-1, which is produced by a filamentous fungus, *Curvularia clavata* (BAUA-2787 strain). KK-1 is probably identical to BK202, which was isolated and purified from *Curvularia* sp. by Novo Nordisk A/S (Bagsværd, Denmark) as described in patent WO1992005191A1 (Nielsen et al., 1992) and is a cyclic depsipeptide composed of one glycine, eight L-amino acids (four valines and one pipecolic acid, aspartic acid, isoleucine, and tyrosine each), and one D-lactic acid. The nitrogen atoms on the amino groups of five of these residues are methylated, and the hydroxy group on the phenyl of the tyrosine residue is also methylated [D-Lac/L-Pip/N-methyl-L-Val/L-Val/N-methyl-L-Asp/N-methyl-L-Val/N-methyl-Ile/Gly/N-methyl-L-Val/O-methyl-L-Tyr] (**Figure 1**, also refer to Yoshimi et al., 2018). KK-1 is a promising pesticide active compound with high activity against many plant pathogens, especially *Botrytis cinerea*, a pathogen that causes grey mold disease in a variety of agricultural and horticultural crops. However, a breakthrough is needed to enable a stable mass supply for practical use. Therefore, we aimed to identify the biosynthetic gene cluster as a first step to the mass production of KK-1 in the future through synthetic biological approaches. The genus *Curvularia*, as well as the genus *Bipolaris*, is a genus of filamentous ascomycetous fungi whose anamorph is *Cochliobolus* (Manamgoda et al., 2011), some of which are known as plant pathogens (Manamgoda et al., 2011), and several bioactive secondary metabolites have been isolated from fungi that belong to these genera (Khiralla et al., 2019; Elkhateeb et al., 2021). However, to date, there have been no reports published on *Curvularia clavata* regarding its ability to produce bioactive compounds or any enzymes related to secondary metabolic pathways, and its genome information has also not been sequenced.

Consequently, in this report, we have conducted whole genome sequencing of *C. clavata* BAUA-2787 as well as transcriptome analysis. Using these data, a gene cluster was identified that is involved in the biosynthesis of KK-1. Then, overexpression or deletion mutants of each cluster gene were generated. We also analyzed the KK-1 production of these mutants and the expression of the cluster genes to demonstrate that these genes are involved in the biosynthesis of KK-1.

## Materials and methods

### Strain and growth condition

*Curvularia clavata* BAUA-2787 was donated by Akita Konno Co., Ltd., Akita, Japan and registered at the National Institute of Technology and Evaluation (No. NITE BP-02399). The conidial suspension of *C. clavata* BAUA-2787 strain was prepared by adding sterile water to the colonies that were grown on CMA medium (Tanaka et al., 1991) [0.15% Ca(NO_3_)_2_·4H_2_O, 0.05% MgSO_4_·7H_2_O, 0.05% KCl, 0.04% KH_2_PO_4_, 0.003% K2HPO4, 0.1% tryptone, 0.1% yeast extract, 1% glucose, and 1.5% agar], scraping them with a sterile spatula, and filtering them through a Miracloth (Calbiochem, San Diego, CA, USA) to remove the mycelium. They were stored in 16% v/v glycerol at -80°C. All *C. clavata* wild-type and mutant strains that were derived from BAUA-2787 were cultured in CM (CMA minus agar) medium that was prepared in Erlenmeyer flasks, and they were inoculated with the conidial suspensions at a concentration of 10^3^ conidia/mL.

### Genome sequencing

The genomic DNA of *C. clavata* BAUA-2787 was extracted using a DNeasy Plant Mini Kit (QIAGEN, Manchester, UK) after 2 days of cultivation in 100 mL CM medium at 26°C and 130 rpm. The DNA libraries were prepared with a 5500 SOLiD Mate-Paired Library Kit and sequenced by a 5500xl SOLiD system (Life Technologies, Carlsbad, CA, USA). A Nextera DNA Sample Prep Kit was also used for the library preparation, and the libraries were sequenced using the MiSeq platform (Illumina, San Diego, CA, USA). The hybrid *de novo* genome assembly (Ikegami et al., 2015) was performed using the MiSeq and SOLiD short read data. Additionally, the protein-coding genes in the draft genome were predicted by a combination of the gene prediction programs, ALN (Gotoh, 2000) and GlimmerHMM (Majoros et al., 2004). NCBI BLAST (http://blast.ncbi.nlm.nih.gov/) and the Pfam protein family database (http://pfam.xfam.org/) were used for sequence homology and conserved domain searches, as appropriate.

### Search for KK-1 biosynthetic non-ribosomal peptide synthetase gene

A BlastP search was performed against the database of the amino acid sequences of the predicted proteins that was constructed from the genome information of *C. clavata* BAUA-2787. The sequences of *NPS1-NPS12* were used as a query, which are non-ribosomal peptide synthetases (NRPSs) that were found in *Cochliobolus heterostrophus*, and some highly homologous genes were extracted. To predict more accurate coding sequences (CDSs) of these genes, a BlastX search was performed against the GenBank database using nucleic acid sequences from 3,000 bp upstream of the start codon to 3,000 bp downstream of the termination codon of these genes as queries. Then, the domain structures of the proteins that are encoded by these genes were predicted using InterProScan (http://www.ebi.ac.uk/InterProScan/) and antiSMASH (http://antismash.secondarymetabolites.org) to search for the NRPSs with a modular structure that is consistent with the structural features of KK-1.

### Prediction of KK-1 biosynthetic gene cluster based on gene expression levels

Wild-type strains of *C. clavata* BAUA-2787 were cultured in 100 mL of CM medium that was prepared in a 300 mL Erlenmeyer flask at 130 rpm for 48 hours at two temperature conditions (26°C and 37°C). The total RNA of these samples was extracted with ISOGEN (Nippon Gene, Toyama, Japan) and purified using the RNeasy spin column (QIAGEN). Then, the quantity and purity of the extracted RNA were determined with an Agilent 2100 Bioanalyzer (Agilent Technologies, Palo Alto, CA, USA). The RNA sequencing (RNA-Seq) libraries were prepared with a Truseq RNA Sample Prep Kit v2 (Illumina) and RNA-Seq was performed on a Miseq system (paired-end 2 × 75 bases). The obtained reads were mapped onto the draft genome of *C. clavata* BAUA-2787 using the TopHat program (Trapnell et al., 2009), and the reads per kilobase of exon per million mapped reads (RPKM) values were calculated from the raw read count data for every gene on the genome. The novel gene cluster mining tool, motif-independent *de novo* detection algorithm for secondary metabolite gene clusters (MIDDAS-M), which predicts co-regulated gene clusters using genome and transcriptomic data (Umemura et al., 2013), was then used to detect the significantly expressed gene clusters at 26°C when compared to 37°C.

### Drug susceptibility of *C. clavata* BAUA-2787 protoplasts

We prepared 5 µL of the protoplast suspension of *C. clavata* BAUA-2787 wild-type strain according to the transformation method as described below. It was spotted in the center of CM-sucrose agar medium (CM medium containing 1.5% agar and 1.2M sucrose) supplemented with six concentrations (1, 2.5, 5, 10, 15, and 20 µg/mL) of aureobasidin A (AbA; Takara Bio, Shiga, Japan) or six concentrations (12.5, 25, 50, 100, 200, and 400 µg/mL) of hygromycin (Hyg; Fujifilm Wako Pure Chemical, Osaka, Japan). After 6 days of incubation at 26 ˚C, the mycelial growth inhibition was evaluated.

### Transformation of *C. clavata* BAUA-2787 with pAUR316

Wild-type strains of *C. clavata* BAUA-2787 were cultured in 100 mL of CM medium, which was prepared in a 300 mL Erlenmeyer flask at 30 ˚C and 130 rpm. After 40 hours, the mycelia were collected by filtration using a Miracloth, washed with sterilized water, and pressed with a spatula to lightly remove the water. The washed mycelia were suspended in 10 mL of protoplast forming solution [3 mg/mL Yatalase (Takara Bio), 0.3 mg/mL Lysing Enzymes from *Trichoderma harziaum* (Sigma-Aldrich, Steinheim am Albuch, Germany), 0.8 mol/L NaCl, and 10 mM sodium phosphate buffer (pH6.0)] and gently shaken at 30 ˚C and 80 rpm. After 3 hours, the cell wall debris was removed by filtration using a Miracloth, and the protoplasts were collected by centrifuging the flow-through. Next, the protoplast pellet was washed with 0.8 mol/L NaCl twice, suspended in Solution 1 [0.8 mol/L NaCl, 10 mM CaCl_2_, and 10 mM Tris-HCl buffer (pH8.0)] to 2 × 10^8^ protoplasts/mL, and 1/5 volume of Solution 2 [40% (w/v) PEG4000, 50 mM CaCl_2_, and 50 mM Tris-HCl buffer (pH8.0)] was added with gentle mixing. Approximately 10 µg of pAUR316 (Takara Bio) was added to a 0.2 mL aliquot of the protoplast suspension, and this was incubated on ice for 10 min. Subsequently, 1 mL of Solution 2 was added to this suspension and incubated at room temperature for 15 min. Afterward, 0.2 mL of the suspension was mixed with 7 mL CM-sucrose top agar medium (CM medium containing 1% agar and 1.2M sucrose) with 10 μg/mL of AbA, and it was overlaid on the surface of the CM-sucrose agar medium (CM medium containing 1.5% agar and 1.2M sucrose) with 10 μg/mL of AbA prepared in petri dishes. These were incubated at 26°C until the colonies of the transformants appeared. Also, direct polymerase chain reaction (PCR) using a KAPA3G Plant PCR Kit (Kapa Biosystems, Wilmington, MA, USA) was performed on the growing colonies to confirm if they harbored pAUR316. The primer information is shown in **Supplementary Table S1**.

### Construction of the DNA fragment for *CcpyrG* Deletion

A Blastp search was performed using the amino acid sequence of the protein that is encoded by *pyrG* of *Aspergillus nidulans* (accession: AAB66359) as the query, and a gene that was found to be a homolog in *C. clavata* BAUA-2787 was designated as *CcpyrG*. The region from 2,005 bp upstream of the start codon to 1,261 bp downstream of the stop codon of *CcpyrG* was amplified from the genomic DNA of *C. clavata* BAUA-2787 using a primer set, CcPyrG-del_FW3/CcPyrG-del_RV3, and Phusion Hot Start II High-Fidelity DNA Polymerase (Thermo Fisher Scientific, Waltham, MA, USA). This fragment was phosphorylated by T4 polynucleotide kinase (Toyobo, Osaka, Japan) and ligated using T4 DNA ligase (Nippon Gene) in the pUC18 plasmid that was digested by SmaI (Takara Bio) and dephosphorylated by *E. coli* Alkaline Phosphatase (Toyobo) to construct the plasmid pUC18-*CcpyrG*. Subsequently, an inverse PCR using a primer set, CcPyrG-del_FW2/CcPyrG-del_RV2, and Phusion Hot Start II High-Fidelity DNA Polymerase was performed to amplify the fragment from pUC18-*CcpyrG* that was lacking the sequence from the *CcpyrG* promoter region to the *CcpyrG* terminator region. The fragment was phosphorylated by T4 polynucleotide kinase and self-ligated by T4 DNA ligase to construct the *CcpyrG* deletion plasmid. The DNA cassette, equivalent to the fragment consisting of the 1,150 bp fragment corresponding to the region from 2,005 bp to 856 bp upstream of the start codon of *CcpyrG* and the 1,079 bp fragment corresponding to the region from 183 bp to 1,261 bp downstream of the stop codon, which were directly connected to each other as homology arms for homologous recombination, was amplified from the *CcpyrG* deletion plasmid, using the primer set, CcPyrG-del_FW3/CcPyrG-del_RV3, and Phusion Hot Start II High-Fidelity DNA Polymerase. This DNA cassette was used for the generation of the *CcpyrG* deletion strain. The scheme described above is shown in **Supplementary Figure S1** and the primer information is shown in **Supplementary Table S1**

### Generation of *CcpyrG* deletion strain as a host for transformation

Transformation was basically performed according to the method of introducing pAUR316 which was described above, but 0.2% (w/v) of uridine, 0.02% (w/v) of uracil, and 1 mg/mL of 5-fluoroorotic acid (Fujifilm Wako Pure Chemical) were added to the CM-sucrose top agar medium and CM-sucrose agar medium, respectively, instead of AbA. Then, a direct PCR using a KAPA3G Plant PCR Kit was conducted on the growing colonies to confirm that the target region containing *CcpyrG* was deleted by homologous recombination. The primer information is shown in **Supplementary Table S1**.

### Plasmid construction for overexpression of *kk1F*

The plasmid for the overexpression of *kk1F* gene was constructed by inserting the four DNA fragments in the following order: the 1,000 bp region upstream of the start codon of *Ccnmt1*, which encodes a homolog of Nmt1 from *Schizosaccharomyces pombe*, as its promoter, the cDNA sequence of *kk1F*, the 355 bp region downstream of the stop codon of *Ccnmt1* as its terminator, and *aurA^r^*, an AbA resistance gene that was isolated from *Aspergillus nidulans*, as a selectable marker in the pUC19 plasmid, using an In-Fusion HD Cloning Kit (Clontech, Mountain View, CA, USA). The promoter and terminator were amplified from the genomic DNA of *C. clavata* BAUA-2787, *kk1F* was amplified from the cDNA library of *C. clavata* BAUA-2787, and *aurA^r^* was amplified from a pAUR316 plasmid. The PCR was performed using Phusion Hot Start II High-Fidelity DNA Polymerase. The primer information is shown in **Supplementary Table S1**.

### Generation of *kk1F* overexpression strain

Transformation was basically performed according to the method of introducing pAUR316 as described above. A direct PCR using the KAPA3G Plant PCR Kit (Kapa Biosystems) was performed on the growing colonies to confirm that the target sequence was introduced into the chromosome. The scheme described above is shown in **Supplementary Figure S2**, and the primer information is shown in **Supplementary Table S1**.

### Plasmid construction for deletion of each cluster gene

The plasmid for each cluster gene deletion except *kk1B* gene was constructed by inserting the three DNA fragments in the following order: an approximately 1 kbp fragment that was homologous to the upstream region of the target gene as the homology left-arm, a 2,231 bp fragment corresponding to the region from 853 bp upstream of the *CcpyrG* start codon to 181 bp downstream of its stop codon as the *CcpyrG* selectable marker, and an approximately 1 kbp fragment homologous to the downstream region of the target gene as the homology right-arm in the pUC19 plasmid, using an In-Fusion HD Cloning Kit. The plasmid for *kk1B* deletion was also basically constructed using the same scheme as above but for the homology right-arm, the region from 4,482 bp to 5,463 bp downstream of the start codon of *kk1B*, corresponding to the inside of the open reading frame (ORF), was used. These DNA fragments were amplified from the genomic DNA of *C. clavata* BAUA-2787 using Phusion Hot Start II High-Fidelity DNA Polymerase. Each gene deletion plasmid was linearized by the appropriate restriction enzyme digestion and used for transformation. The primer information is shown in **Supplementary Table S1**, and the region of homology arms that was included in each deletion plasmid and the restriction enzymes that were used for the linearization of each plasmid are listed in **Supplementary Table S2**.

### Generation of each cluster gene deletion strain

Transformation was basically performed according to the method of introducing pAUR316 as described above but the *CcpyrG* deletion strain was used as the host, the CM medium containing 0.2% (w/v) uridine and 0.02% (w/v) uracil was used during the initial culture process, and the CM-sucrose top agar medium and CM-sucrose agar medium without any addition were used for the transformant selection process. A direct PCR using the KAPA3G Plant PCR Kit was performed on the growing colonies to confirm that each cluster gene was deleted by homologous recombination and the transformants were obtained as homokaryons. The scheme described above is shown in **Supplementary Figures S3, S4**, and the primer information is shown in **Supplementary Table S1**.

### Qualitative evaluation of the KK-1 productivity

A small amount of the culture supernatant was sterilized by filtration through a 0.22 µm membrane filter (Merck KGaA, Darmstadt, Germany), and paper disks with a diameter of 6 mm (Advantec, Tokyo, Japan, ø6 mm) were soaked in the supernatant. This paper disk was placed near the center of a potato dextrose agar plate in a 90 mm petri dish and *B. cinerea* was inoculated onto its opposite side. After 3 days of incubation at 26 ˚C, the KK-1 productivity was evaluated based on its effect on the growth of *B. cinerea*. The culture supernatant of the wild-type strain was used as a positive control, and the CM medium was used as a negative control.

### Quantification of KK-1

*C. clavata* BAUA-2787 strains were grown on CM medium prepared in baffled Erlenmeyer flasks, the same volume of acetone was added, and it was sonicated. The sample was then extracted twice with the same volume of ethyl acetate. Then, the ethyl acetate layer was collected, dehydrated with anhydrous sodium sulfate, and evaporated. The extracts were dissolved in acetonitrile and analyzed by ultra-performance liquid chromatography (UPLC), high-performance liquid chromatography (HPLC), or HPLC-mass spectrometry (HPLC-MS). KK-1 was detected at a wavelength of 195 nm. The production of KK-1 was quantified by calculating the peak area of each UV chromatogram. The KK-1 that was isolated and purified from the culture medium of the wild-type strain was used for external calibration.

First, the UPLC measurements were carried out on an ACQUITY UPLC I-Class system (Waters, Milford, MA, USA) and performed using an ACQUITY UPLC BEH C18, 130 Å, 1.7 µm, 2.1 mm x 100 mm column (Waters) at a flow rate of 0.6 mL/min and using a gradient from 50% to 98% acetonitrile in the water containing 0.1% formic acid for 3 min. Second, the HPLC measurements were carried out on a Nexera-i LC-2040C HPLC system (Shimadzu, Kyoto, Japan) and performed using a CAPCELL PAK C18, SG120 Å, 5 µm, 4.6 mm x 250 mm column (Osaka Soda, Osaka, Japan) at a flow rate of 1.0 mL/min and using a gradient from 50% to 98% acetonitrile in water containing 0.1% formic acid for 15 min. Third, the HPLC-MS measurements were carried out on an ExionLC system (AB Sciex, Tokyo, Japan) coupled with a Triple QUAD 4500 system (AB Sciex). The HPLC was performed using a Kinetex 2.6 µm C18, 100 Å, LC column (150 x 4.6 mm; Phenomenex, Torrance, CA, USA) at a flow rate of 0.4 mL/min and using a gradient from 50% to 98% acetonitrile in water containing 0.1% formic acid for 8 min. The MS conditions were as follows: ionization mode, ESI; polarity, positive; turbo gas temperature, 300 ˚C; ion spray voltage, 5.5 kV; and scan range, m/z 500 to 1200.

### RNA-Seq for *kk1F* mutants

The *kk1F* overexpression strain, its deletion strain, and the wild-type strain were cultured on 30 mL of CM medium, which was prepared in a 100 mL Erlenmeyer flask at 26 ˚C and 130 rpm, and RNA-Seq was performed as described above.

## Results

### Search for KK-1 biosynthetic non-ribosomal peptide synthetase gene

In many peptide secondary metabolites, the peptide backbone is known to be synthesized by non-ribosomal peptide synthetase (NRPS). NRPSs are large enzyme complexes that have modular structures, each containing multiple functional domains with specific activities and being capable of catalyzing the synthesis of peptide chains without depending on ribosomal machinery (Hur et al., 2012; Duban et al., 2022). Each module basically contains an adenylation domain (A domain), peptidyl carrier protein domain (PCP domain), and condensation domain (C domain), which are essential for the synthesis of the peptide backbone, and a thioesterase domain (TE domain) that is generally located at the C-terminus in the final module of NRPS and is responsible for the cyclization of the peptide chain (Hur et al., 2012; Duban et al., 2022). The other domains have the function of modifying the peptide backbone that is synthesized by these essential domains (Hur et al., 2012; Duban et al., 2022). Since the order and number of the module structures and the characteristics of their constituent domains are consistent with those of amino acid residues of the corresponding peptide, it is possible to presume that NRPS is involved in the biosynthesis of peptide secondary metabolites based on this information. Therefore, we searched for the NRPS gene that is responsible for the biosynthesis of the peptide backbone of KK-1 as a first step to identify the biosynthetic gene cluster in *C. clavata* genome.

**Figure 2:**
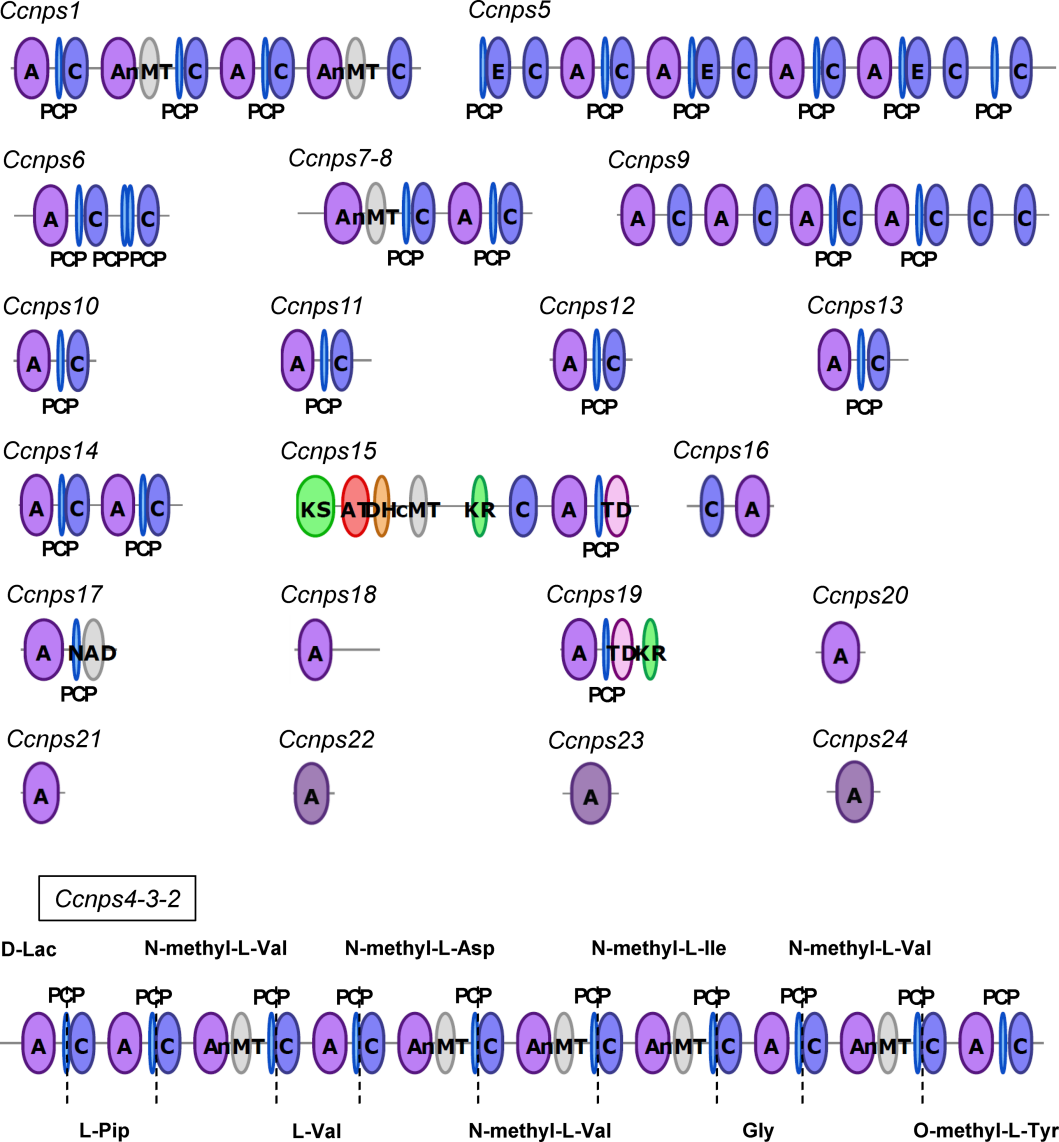
The domain structure of each non-ribosomal peptide synthetase that is possessed by *Curvularia clavata* BAUA-2787, which was deduced by antiSMASH: C, condensation domain; A, adenylation domain; PCP, peptidyl carrier protein domain; nMT, nitrogen methyl transferase; E, epimerization domain; KS, ketosynthase domain; AT, acyltransferase domain; DH, dehydratase domain; cMT, carbon methyltransferase; KR, ketoreductase domain; TD, terminal reductase domain; NAD, male sterility protein. Only the modular structure of NRPS that is encoded by *Ccnps4-3-2* is consistent with the structural characteristics of KK-1.

We initially performed draft genome sequencing of *C. clavata* BAUA-2787 and obtained 142 sequences with an N50 value of 1,472,240 bp for a total of 30.6 Mbp at 50.3% GC content, as a result of the hybrid assembly combining the SOLiD and Illumina data. A total of 10,367 ORFs were predicted from this draft genome sequence. Subsequently, we searched the draft sequence for the genes encoding NRPS. In *Cochliobolus heterostrophus*, which is closely related to *C. clavata*, 12 NRPS genes (*NPS1*–*NPS12*) have been found (Lee et al., 2005). A BlastP search against the sequence information of the predicted proteins found in *C. clavata* BAUA-2787 was carried out using the sequences of *NPS1*–*NPS12* as queries, resulting in 24 ORFs as candidates of the NRPS gene that is responsible for KK-1 synthesis, which were designated as *Ccnps1*–*Ccnps24*. The more accurate sequences of each of these ORFs were predicted by a homology search against the GenBank database, and it was shown that *Ccnps4, Ccnps3, and Ccnps2* were one gene connected in this order. We called the gene *Ccnps4-3-2.* To find the NRPS gene that was consistent with the structural features of the cyclic peptide that is composed of 10 residues of KK-1, the domain structures of the proteins that were encoded by the above ORFs were subsequently examined using antiSMASH (**Figure 2**). Consequently, only *Ccnps4-3-2* with a total length of 39,125 bp had 10 modules, each of which contained A, PCP, and C domains, suggesting that it is the NRPS gene that is involved in the synthesis of KK-1. In addition, the NRPS that is encoded by *Ccnps4-3-2* has five N-methyltransferase domains (nMT domains), responsible for N-methylation of the amino acids that are incorporated into the same module and are located in the third, fifth, sixth, seventh, and ninth modules from the N-terminus, respectively. If the first module of the NRPS that is encoded by *Ccnps4-3-2* is involved in the introduction of the L-lactic acid residue of KK-1, the order of the modules that contained the nMT domains perfectly matched that of the N-methylated amino acid residue in KK-1 (**Figure 2**). This also strongly suggests that *Ccnps4-3-2* is probably the NRPS that is responsible for the synthesis of the basic peptide backbone of KK-1.

### Prediction of KK-1 biosynthetic gene cluster based on gene annotation

**Figure 3:**
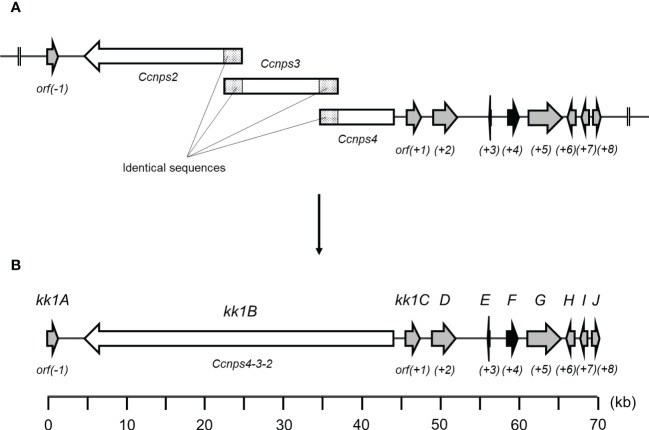
**(A)** A fragmentation image of *Ccnps4-3-2*, namely *kk1B*, into three ORFs (*Ccnps2*, *Ccnps3*, and *Ccnps4*) using the obtained sequencing data of *Curvularia clavata* BAUA-2787. **(B)** The genetic map and predicted gene arrangement of the KK-1 biosynthetic gene cluster (see **Table 2**). The gene *kk1B*, encoding non-ribosomal peptide synthetase (NRPS), is shown as a white arrow, and *kk1F*, encoding the transcription factor, is shown as a black arrow.

In general, genes that are involved in the biosynthesis of secondary metabolites exist as clusters. For cyclosporine, which is used as an immunosuppressant agent, a series of genes including *simA*, which encodes the NRPS responsible for the synthesis of the basic backbone, has been identified as a putative biosynthetic gene cluster in an approximately 95 kb region of the *Tolypocladium inflatum* genome (Bushley et al., 2013). For daptomycin, a series of genes including three NRPS genes (*dptA*, *dptBC*, and *dptD*), which are essential for the synthesis of the basic backbone, are located at the same locus on the genome of *Streptomyces roseosporus*, and these genes are known to be involved in the biosynthesis of this antibiotic (Miao et al., 2005). Based on these cases, a group of KK-1 biosynthetic genes could also be present as a gene cluster containing *Ccnps4-3-2*. Therefore, for the 14 ORFs that were found upstream of *Ccnps4-3-2* (designated *orf(-14)*–*orf(-1)*) and the other 14 ORFs found downstream (designated *orf(+1)*–*orf(+14)*), the function of the protein that is encoded by each gene was predicted (**Table 1**), and the region of approximately 71 kb containing a series of 10 genes from *orf(-1)* to *orf(+8)* that are likely to be involved in secondary metabolism was predicted as the KK-1 biosynthetic gene cluster (**Figure 3A**). These 10 genes are hereinafter referred to as *kk1A*–*kk1J*, and the NRPS gene, *Ccnps4-3-2*, corresponds to *kk1B*. About half of the region of the cluster was occupied by *kk1B* (**Figure 3B**; **Table 2**). The *kk1F* gene was predicted to encode a protein with a basic-region leucine zipper (bZIP) motif. This is the only putative transcription factor gene that was found in the cluster, and, therefore, was thought to be important in regulating the expression of the other cluster genes. The accuracy of the KK-1 cluster region assembly was later confirmed by performing long read sequencing with PacBio RS-II (data not shown). The 75 kb sequence that encompasses the above cluster region has been deposited in GenBank (accession: LC371755).

**Table 1:** *C. clavata* BAUA-2787 open reading frames (ORFs) around *Ccnps4-3-2*.

ORF	Protein Length (aa)	Genbank homolog	Accession no.	E-value	Conserved domain in Pfam database (Significant Pfam-A matches)
Upstream region					
*orf(-14)*	247	ubiquinol-cytochrome-c reductase, *Stemphylium lycopersici*	KNG45667.1	3E-98	ND
*orf(-13)*	68	hypothetical protein/uncharacterized protein			ND
*orf(-12)*	335	kinesin light chain 3, *Pyrenophora tritici-repentis* Pt-1C-BFP	XP_001941711.1	3E-173	PF13374:Tetratricopeptide repeat PF13424:Tetratricopeptide repeat
*orf(-11)*	551	kinesin light chain 3, *Paraphaeosphaeria sporulosa*	XP_018040440.1	0.0	PF00931:NB-ARC domain PF06985:Heterokaryon incompatibility protein (HET)
*orf(-10)*	325	Putative Transposase, *Penicillium brasilianum*	CEJ62693.1	8E-169	PF13358:DDE superfamily endonuclease
*orf(-9)*	253	Cytochrome P450, *Pyrenophora seminiperda* CCB06	RMZ67022.1	2E-130	PF17111:Fungal N-terminal domain of STAND proteins
*orf(-8)*	798	TPR-like protein, *Pyrenochaeta* sp. DS3sAY3a	OAL55283.1	0.0	PF00931:NB-ARC domain PF13374:Tetratricopeptide repeat PF13424:Tetratricopeptide repeat
*orf(-7)*	201	HET-domain-containing protein, *Glonium stellatum*	OCL05376.1	2E-57	PF06985:Heterokaryon incompatibility protein (HET)
*orf(-6)*	105	HET-domain-containing protein, *Glonium stellatum*	OCL05376.1	2E-37	ND
*orf(-5)*	242	ankyrin, partial, *Cenococcum geophilum* 1.58	OCK95360.1	4E-89	PF12796:Ankyrin repeats (3 copies) PF13637:Ankyrin repeats (many copies)
*orf(-4)*	166	subtilisin-like serine protease, *Stemphylium lycopersici*	KNG48874.1	4E-78	PF20246:Family of unknown function (DUF6601)
*orf(-3)*	1014	TPR-like protein, *Corynespora cassiicola*	PSN58929.1	0.0	PF00931:NB-ARC domain PF13424:Tetratricopeptide repeat
*orf(-2)*	212	hypothetical protein/uncharacterized protein			ND
Putative KK-1 cluster					
*orf(-1)/kk1A*	422	S-adenosyl-L-methionine-dependent methyltransferase, *Trematosphaeria pertusa*	XP_033691074.1	0.0	PF00891:O-methyltransferase domain
*Ccnps4-3-2/kk1B*	13041	cyclosporine synthetase, *Dactylonectria estremocensis*	KAH7155895.1	0.0	PF00501:AMP-binding enzyme PF00550:Phosphopantetheine attachment site PF00668:Condensation domain PF13193:AMP-binding enzyme C-terminal domain PF13649:Methyltransferase domain
*orf(+1)/kk1C*	538	amidase, Cenococcum geophilum 1.58	OCK86552.1	0.0	PF01425:Amidase
*orf(+2)/kk1D*	996	hypothetical protein/uncharacterized protein			ND
*orf(+3)/kk1E*	77	gdp-mannose transporter, partial, *Colletotrichum incanum*	KZL70657.1	2E-15	ND
*orf(+4)/kk1F*	398	bZIP transcription factor, *Beauveria felina*	ATQ39425.1	3E-18	ND
*orf(+5)/kk1G*	1338	Multidrug resistance protein 2, *Alternaria arborescens*	XP_028500901.1	0.0	PF00005:ABC transporter, nucleotide-binding domain PF00664:ABC transporter transmembrane region
*orf(+6)/kk1H*	348	d-lactate dehydrogenase, *Quercus suber*	POF13829.1	1E-177	PF00389:D-isomer specific 2-hydroxyacid dehydrogenase, catalytic domain PF02826:D-isomer specific 2-hydroxyacid dehydrogenase, NAD binding domain
*orf(+7)/kk1I*	311	pyrroline-5-carboxylate reductase, *Periconia macrospinosa*	PVH93517.1	1E-146	PF14748:Pyrroline-5-carboxylate reductase dimerisation PF03807:NADP oxidoreductase coenzyme F420-dependent
*orf(+8)/kk1J*	307	alpha/beta-Hydrolase, *Glarea lozoyensis* ATCC 20868	XP_008084349.1	3E-88	PF00975:Thioesterase domain
Downstream region					
*orf(+9)*	175	hypothetical protein/uncharacterized protein			ND
*orf(+10)*	385	gdp-mannose transporter, partial, *Colletotrichum incanum*	KZL70657.1	0.0	ND
*orf(+11)*	183	AC transposase, *Stemphylium lycopersici*	RAR14477.1	1E-41	ND
*orf(+12)*	300	Dimer-Tnp-hAT domain-containing protein, *Pyrenophora tritici-repentis*	KAA8617358.1	5E-20	ND
*orf(+13)*	137	Histone H3.3, *Rozella allomycis* CSF55	EPZ32225.1	6E-43	PF00125:Core histone H2A/H2B/H3/H4
*orf(+14)*	284	ATP binding protein, *Alternaria alternata*	OWY49600.1	1E-103	PF00145:C-5 cytosine-specific DNA methylase

ND, not detected.

**Table 2:** Genetic locus information in the KK-1 cluster.

ORF	Strand	Start	End	Length (bp)	Gene name in GenBank (Protein ID)
*kk1A*	+	1	1,373	1,373	TRAF135002 (BBC83956.1)
*kk1B*	–	4,746	43,871	39,126	TRAF135001 (BBC83957.1)
*kk1C*	+	45,394	47,265	1,872	TRAF068002 (BBC83958.1)
*kk1D*	+	48,867	51,857	2,991	TRAF068003 (BBC83959.1)
*kk1E*	–	55,802	56,035	234	TRAF068004 (BBC83960.1)
*kk1F*	+	58,277	59,633	1,357	TRAF068005 (BBC83961.1)
*kk1G*	+	61,478	65,761	4,284	TRAF068006 (BBC83962.1)
*kk1H*	–	66,348	67,394	1,047	TRAF068007 (BBC83963.1)
*kk1I*	–	68,169	69,104	936	TRAF068008 (BBC83964.1)
*kk1J*	+	69,822	70,967	1,146	TRAF068009 (BBC83965.1)

### Prediction of KK-1 biosynthetic gene cluster based on gene expression levels

**Figure 4:**
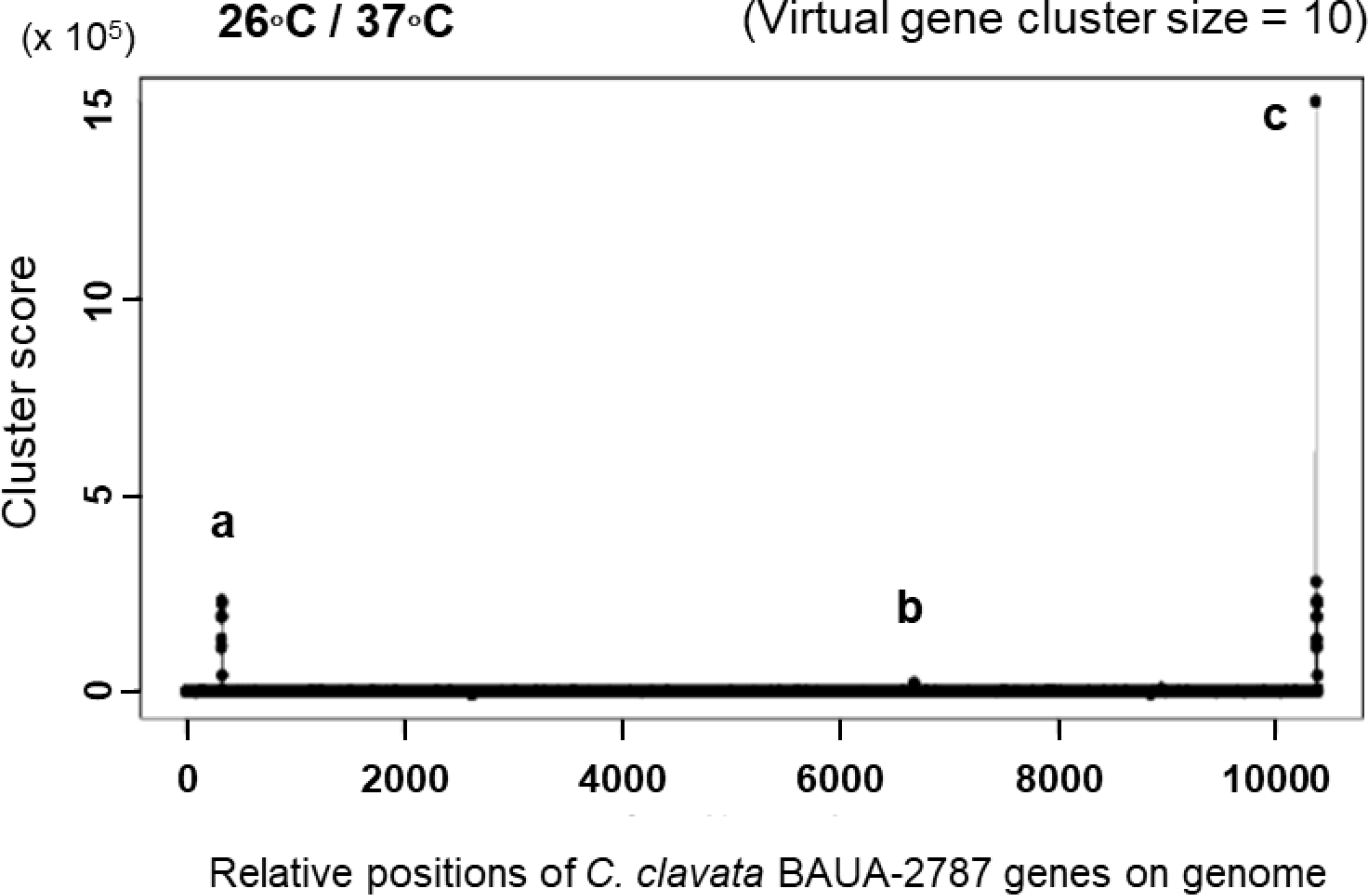
KK-1 cluster detection by MIDDAS-M. The gene clusters whose expression levels increased at 26 ˚C, the KK-1-producing culture temperature condition, when compared to 37˚C, the non-producing culture temperature condition. The analysis was performed on the data that consisted of the manually connected sequence of the putative KK-1 cluster, which was presumed to have been fragmented into three sequences (see **Figure 3**) and is at the end of all the obtained gene data at the right end of the horizontal axis. The three detected peaks are indicated by **(A–C**, **(A)**, a fragmented KK-1 cluster containing *Ccnps4*; **(B)** a fragmented KK-1 cluster containing *Ccnps2*; and **(C)** a manually connected KK-1 cluster of the three fragments.

Cluster prediction was also performed using MIDDAS-M (Umemura et al., 2013), a sequence motif-independent detection method of secondary metabolism biosynthesis gene clusters. It can sensitively detect the gene clusters in which the genes are cooperatively regulated at transcriptional levels using genome sequence information that is combined with transcriptomic data that are obtained under compound-producing and non-producing conditions. *C. clavata* BAUA-2787 produced KK-1 well on the CM medium at 26 ˚C but less was produced at 37°C. Therefore, we set 26°C as the KK-1 production condition and 37°C as the KK-1 non-production condition, and RNA-Seq data were obtained under both conditions. As a result of cluster identification using MIDDAS-M and these data, a series of genes, including the 10 ORFs that were referred to above, were significantly detected (**Figure 4**), indicating that the expression of these genes was co-induced during KK-1 production. This result supports that this gene cluster is involved in the biosynthesis of KK-1.

### Development of transformation system

To analyze the function of each ORF, we started to develop a transformation system for *C. clavata*, which had not been previously established. Since the protoplast-PEG method had been well known as a transformation method for filamentous fungi, we first examined the conditions for the preparation of protoplasts of *C. clavata* BAUA-2787. As a result, we found that the protoplasts can be efficiently obtained by adding an enzyme solution that contains Yatalase and Lysing Enzymes from *Trichoderma harziaum* to culture mycelia on a 100 mL CM medium that is prepared in a 300 mL Erlenmeyer flask at 30°C and 130 rpm for 40 hours.

**Figure 5:**
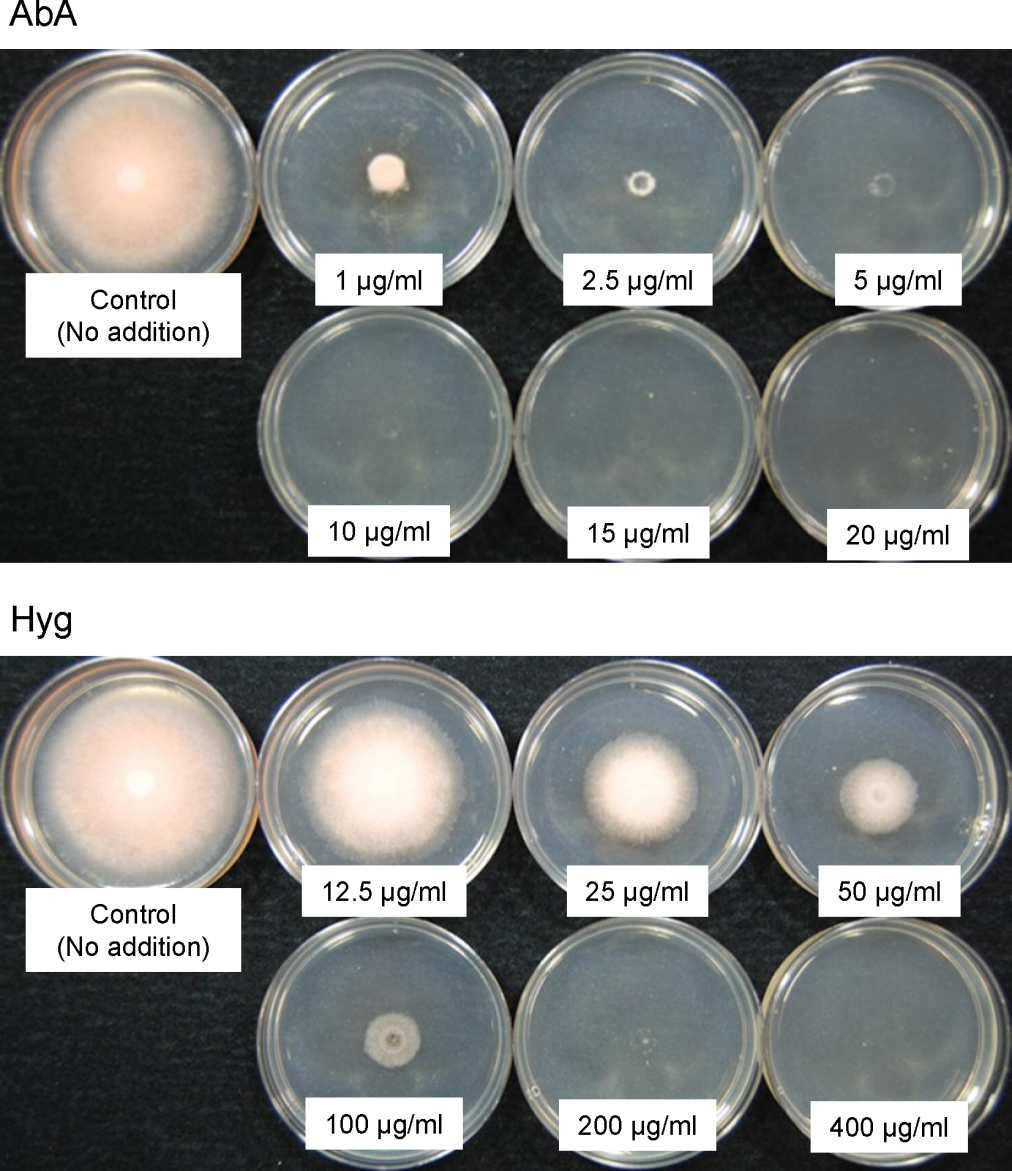
The susceptibility of the *Curvularia clavata* BAUA-2787 protoplast to aureobasidin A (AbA) and hygromycin (Hyg). The concentration of each agent in the medium is shown in the pictures.

Next, the susceptibility of *C. clavata* BAUA-2787 protoplasts to AbA and Hyg was examined on the CM-sucrose agar medium to find drug-selectable markers for transformation. The results showed that AbA and Hyg almost completely inhibited mycelial growth from the protoplasts at concentrations of 10 μg/mL or higher and 200 μg/mL or higher, respectively (**Figure 5**), suggesting that these concentrations can be used to select transformants that confer resistance to the corresponding drugs.

**Figure 6:**
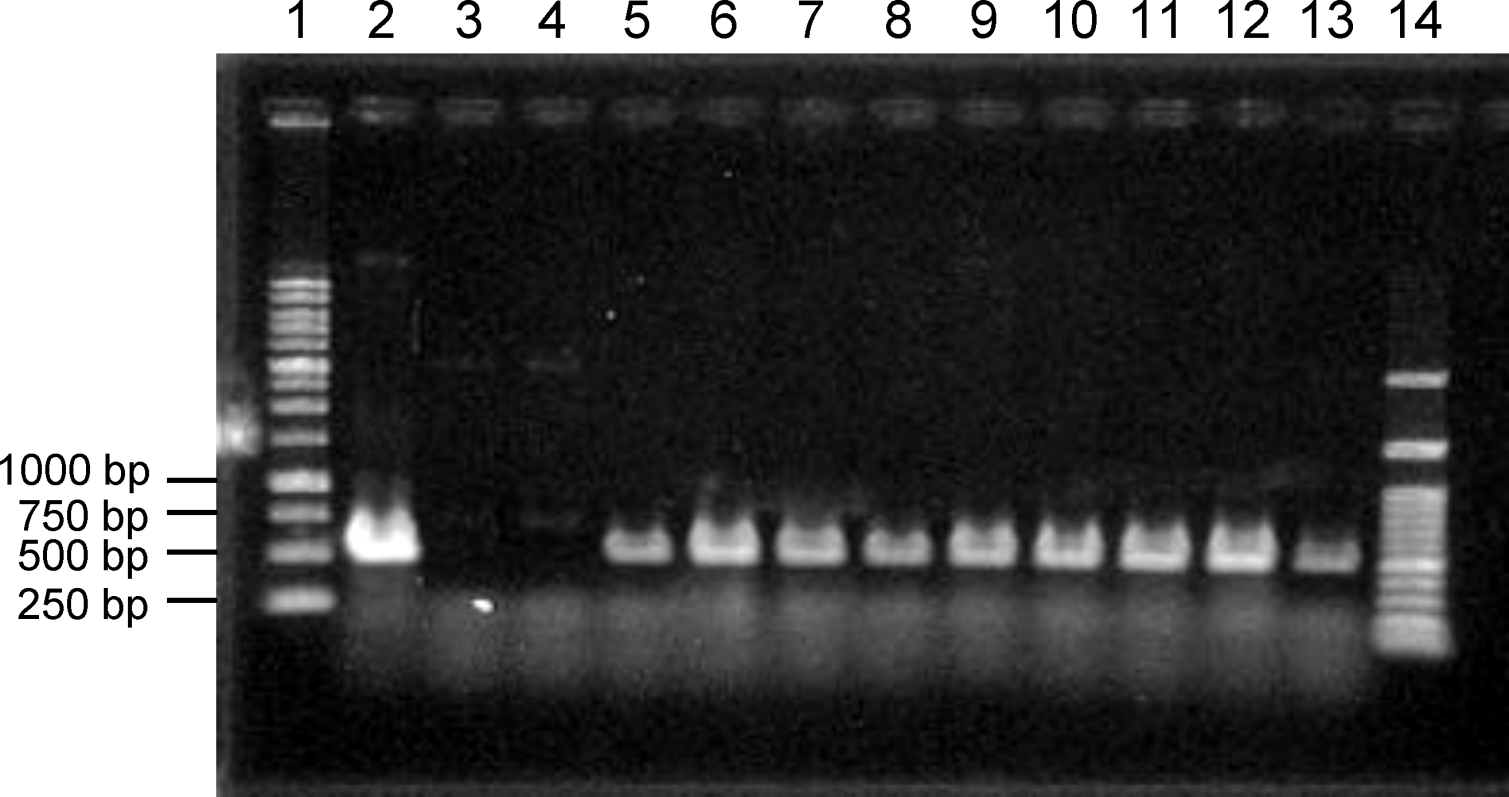
Agarose gel electrophoresis to confirm the introduction of pAUR316 into *Curvularia clavata* BAUA-2787. Lane 1, 1 kbp DNA ladder; lane 2, pAUR316 plasmid; lane 3, wild-type strain as the negative control; lane 4, non-transformant (negative result); lane 5-13, pAUR316 transformants; and lane 14, 100 bp DNA ladder.

With the necessary conditions in place, as described above, we attempted to transform *C. clavata* BAUA-2787 using the protoplast-PEG method and the pAUR316 plasmid, which is known as a shuttle vector for *Aspergillus* and contains the AbA resistance gene *aurA* ^r^ from *Aspergillus nidulans* as a selectable marker. Consequently, strains that could grow on the CM-sucrose agar medium containing 10 μg/mL AbA were obtained, and these strains also showed good growth when transferred to the CMA medium containing 2.5 μg/mL AbA. Furthermore, a PCR to amplify a partial sequence of pAUR316 revealed that these strains harbor pAUR316 (**Figure 6**), indicating that *aurA* ^r^ carried functions also in *C. clavata* BAUA2787, and the transformation system using AbA was successfully developed.

### Generation of *pyrG* deletion strain as a host of transformation

In filamentous fungi, the *pyrG* gene, which is homologous to the *Saccharomyces cerevisiae* URA3 gene, is important for uridine synthesis and has often been used as a selectable auxotrophic marker for transformation (Van Hartingsveldt et al., 1987; Ling et al., 2013). In this study, the use of the *pyrG* homolog as an alternative selectable marker to the drug-resistant markers in *C. clavata* BAUA-2787 was examined. The *pyrG* gene encodes orotidine-5’-phosphate decarboxylase, which is responsible for the conversion of orotidine monophosphate to uridine monophosphate in the pyrimidine biosynthetic pathway, and the mutants lacking this function are auxotrophic for uridine and uracil and are also resistant to 5-fluoroorotic acid (5-FOA). A BlastP search revealed the gene which shows 55% similarity to the protein sequence that is encoded by *pyrG* of *Aspergillus nidulans*, and this gene was hereafter referred to as *CcpyrG*. Subsequently, to generate a *CcpyrG* deletion strain for use as a host, a *CcpyrG* deletion DNA cassette was prepared and used to transform the wild-type strain of *C. clavata* BAUA-2787 (**Supplementary Figure S1**). Therefore, we succeeded in obtaining the *CcpyrG* deletion strain, in which the DNA region containing *CcpyrG* was deleted by homologous recombination, and it became auxotrophic for uridine and uracil and 5-FOA-resistant.

### Analysis of *kk1F* overexpression strain

Since *kk1F*, found in the putative biosynthetic gene cluster of KK-1, was predicted to be a transcription factor and was considered to regulate the expression of the other genes in the cluster, *kk1F* mutants were generated to analyze the function of this gene. Initially, the overexpression strain of *kk1F* was generated. As a promoter for overexpression, we focused on the gene which encodes a homolog of Nmt1 from *Schizosaccharomyces pombe* and Thi5p from *Saccharomyces cerevisiae*, and the gene shows 74% similarity to the conserved region (NMT1/THI5 like domain: Pfam 09084) of Nmt1 (accession: AAA35318) and was designated as *Ccnmt1*. Since the *nmt1* gene has been known to have a strong promoter that can be regulated by thiamine in *S. pombe* (Maundrell, 1990) and *Ccnmt1* is also highly expressed in a constant manner in *C. clavata* BAUA-2787 based on the RNA-Seq data, we decided to overexpress *kk1F* by using the *Ccnmt-1* promoter sequence. Specifically, we constructed a plasmid with a DNA cassette in which *kk1F* was connected to the region that probably contained the *Ccnmt1* promoter, and the wild-type strain of *C. clavata* BAUA-2787 was transformed using the plasmid (**Supplementary Figure S2**). Then, the transformants that were confirmed to have the target sequence introduced onto the chromosome were the *kk1F* overexpression strains.

**Figure 7:**
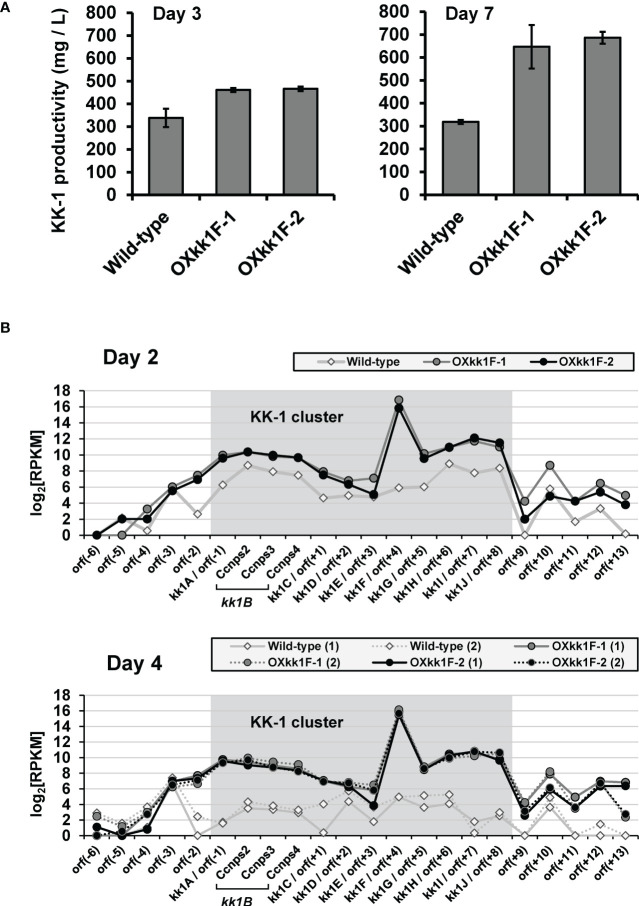
Analysis of *kk1F* overexpression strain. **(A)** The KK-1 productivity of the wild-type and two *kk1F* overexpression strains (OXkk1F-1, OXkk1F-2) on day 3 and day 7 of the culture. The error bars represent standard deviations (three biological replicates). **(B)** The reads per kilobase of exon per million mapped reads (RPKM) expression values in log2 scale for the putative KK-1 biosynthetic gene cluster corresponding to the gene IDs from *kk1A* to *kk1J* that are shown as shading and the surrounding genes in the above three strains on day 2 and day 4 of the culture. The numbers (1) and (2) indicate biological replicates. The region containing *Ccnps2*, *Ccnps3*, and *Ccnps4* corresponds to *kk1B*, which encodes non-ribosomal peptide synthetase.

KK-1 production of these strains was quantified on day 3 and day 7 of the cultures by LC-MS, and it was found to be increased when compared to the wild-type strain production on both days, reaching approximately double on day 7 (**Figure 7A**). Furthermore, the RNA-Seq analysis for day 2 and day 4 of the cultures revealed that the expression levels of the entire putative cluster of the *kk1F* overexpression strains were clearly higher than those of the wild-type strains, and the difference was greater on day 4 than on day 2 (**Figure 7B**). These results strongly suggested that stronger expression of *kk1F* increased the expression of the cluster genes, resulting in increased KK-1 productivity.

It should be noted that the compound corresponding to the peak used as an indicator for the quantification of KK-1 above was isolated and analyzed by infrared (IR) and proton nuclear magnetic resonance (^1^H-NMR) spectroscopy (**Supplementary Figures S5, S6**), and the respective spectra obtained certainly matched those of BK202, which is probably the same compound as KK-1, described in the patent WO1992005191A1 (Nielsen et al., 1992).

### Analysis of *kk1F* deletion strain

A *kk1F* deletion strain was also generated to examine KK-1 production and the cluster gene expression levels. A plasmid was constructed with sequences upstream and downstream of the *kk1F* ORF as homology arms for the deletion of this ORF by homologous recombination, and *CcpyrG*, including its putative promoter and terminator regions, was located between them as a selectable marker. Then, this plasmid, linearized with a restriction enzyme, was used to transform the *C. clavata* BAUA-2787 *CcpyrG* deletion strain (**Supplementary Figure S3**). Accordingly, the transformants that were confirmed to be homokaryons with *kk1F* deleted were the *kk1F* deletion strains.

**Figure 8:**
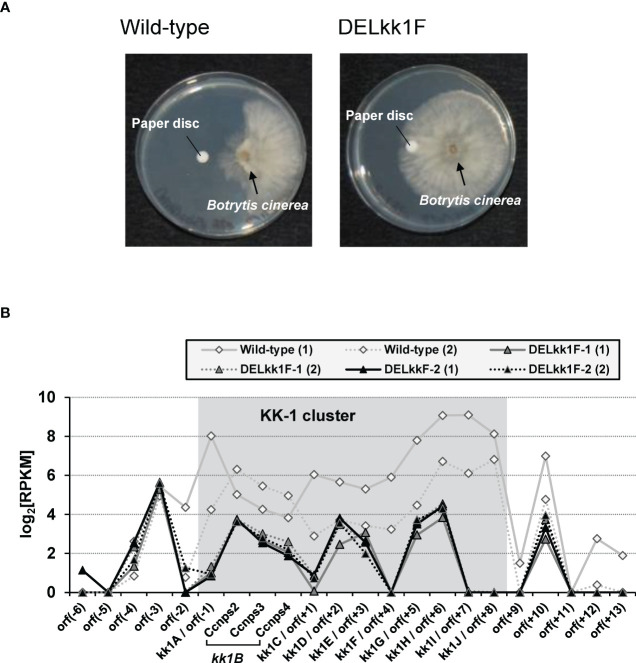
Analysis of the *kk1F* deletion strain. **(A)** Biological assays of the antifungal activity in the culture supernatants of the wild-type and *kk1F* deletion strains (DELkk1F). The pictures show the *Botrytis cinerea* colonies grown against paper discs soaked with the culture supernatant of each strain. **(B)** The reads per kilobase of exon per million mapped reads (RPKM) expression values in log2 scale for the putative KK-1 biosynthetic gene cluster corresponding to the gene IDs from *kk1A* to *kk1J* that are shown as shading and the surrounding genes of the wild-type and two *kk1F* deletion strains (DELkk1F-1, DELkk1F-2) on day 3 of the culture. The numbers (1) and (2) indicate biological replicates. The region containing *Ccnps2*, *Ccnps3*, and *Ccnps4* corresponds to *kk1B*, which encodes non-ribosomal peptide synthetase.

Quantification of KK-1 on day 7 of the culture showed that the *kk1F* deletion strains hardly produced KK-1, and the antifungal activity of their culture supernatant against *B. cinerea* was also removed (**Figure 8A**). Furthermore, the deletion strains showed reduced transcriptional expression levels for the entire putative cluster when compared to those of the wild-type strains (**Figure 8B**). These results suggested that the deletion of *kk1F* led to reduced expression of the cluster genes, resulting in the loss of KK-1 productivity.

### Analysis of each cluster gene deletion strain

To investigate the involvement of the putative cluster genes other than *kk1F* in KK-1 biosynthesis, the deletion mutants of each gene were generated and KK-1 productivity was evaluated. For each gene, a plasmid of almost the same design as that which was used to delete *kk1F* was constructed, and each of these plasmids was linearized by restriction enzymes and transformed into the *C. clavata* BAUA-2787 *CcpyrG* deletion strain (**Supplementary Figures S3, S4**). Thus, the transformants that were confirmed to be homokaryons with a deleted target gene were obtained for each gene deletion strain.

**Figure 9:**
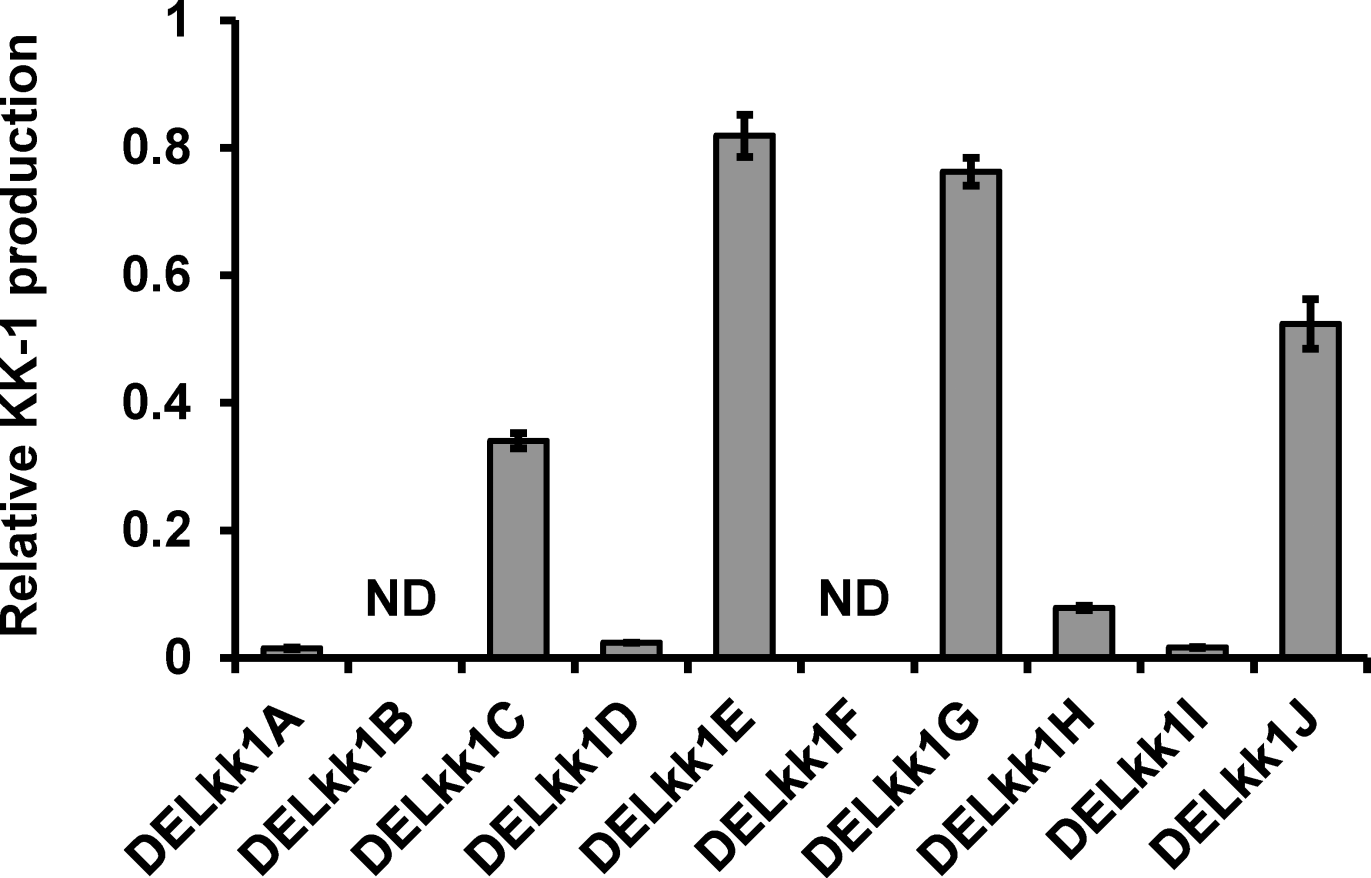
The relative KK-1 production of each cluster gene deletion strain when compared to that of the wild-type strain on day 7 of the culture. The abbreviations for the mutants are shown on the horizontal axis: *kk1A* deletion strain, DELkk1A; *kk1B* deletion strain, DELkk1B; *kk1C* deletion strain, DELkk1C; *kk1D* deletion strain, DELkk1D; *kk1E* deletion strain, DELkk1E; *kk1F* deletion strain, DELkk1F; *kk1G* deletion strain, DELkk1G; *kk1H* deletion strain, DELkk1H; *kk1I* deletion strain, DELkk1I; *kk1J* deletion strain, DELkk1J. The error bars represent standard deviations (three biological replicates). ND, not detected.

The KK-1 production of each mutant was then measured using day 7 of the culture (**Figure 9**). KK-1 production was completely lost in the *kk1B* deletion strain, and in four deletion strains (*kk1A*, *kk1D*, *kk1H*, and *kk1I*) it was markedly reduced to less than 10% when compared to that of the wild-type strain, suggesting that these genes are likely to be directly involved in the biosynthesis of KK-1. Furthermore, KK-1 production in the other four deletion strains (*kk1C*, *kk1E*, *kk1G*, and *kk1J*) was also lower than that in the wild-type strain, although the extent of the reduction was smaller than that of the above six mutants. This suggests that these genes are not essential for KK-1 synthesis but are involved in its production in some way by promoting or assisting productivity.

**Figure 10:**
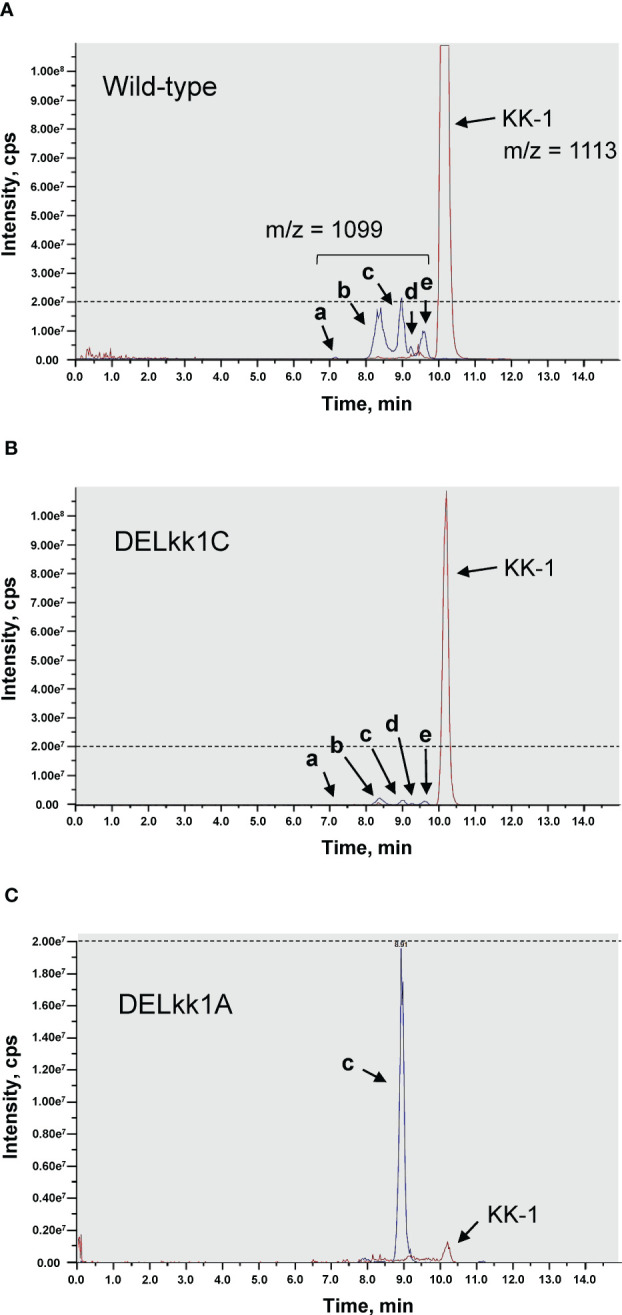
The HPLC-MS analysis of the culture extracts of the *Curvularia clavata* BAUA-2787 strains; **(A)** wild-type strain; **(B)** *kk1C* deletion strain (DELkk1C) as a representative of the KK-1 cluster gene deletion mutants except *kk1A*; and **(C)** *kk1A* deletion stain (DELkk1A). The extracted ion chromatograms are shown for KK-1 ([M+H+] = 1113) and its putative mono-demethylated metabolites (a, b, c, d and e, [M+H+] = 1099). The dotted lines are drawn at the peak intensity of 2.00e7 cps.

To determine whether *kk1A*, predicted to encode an S-adenosyl-L-methionine-dependent methyltransferase, is involved in the synthesis of O-methyltyrosine residues of KK-1, the metabolites in the culture medium of the *kk1A* deletion strain were analyzed and compared with those of the wild-type strain and the other cluster gene deletion strains using LC-MS (**Figure 10**). In addition to the peak of KK-1 (m/z = 1113 [M+H]^+^), five compounds (a, b, c, d, and e) with molecular weights corresponding to the mono-demethylated metabolites of KK-1 (m/z = 1099 [M+H]^+^) were detected in the wild-type strain (**Figure 10A**), all of which were more hydrophilic than KK-1. The production of all of these five compounds was ≤ 1/20 of that of KK-1, and each gene deletion strain basically produced them in proportion to the amount of KK-1 produced (**Figure 10B**). However, even though KK-1 production in the *kk1A* deletion strain was only about 1/100 of that of the wild-type strain, the production of compound c was maintained at the same level as that of the wild-type strain (**Figure 10C**), reversing the abundance ratio of KK-1 to compound c.

## Discussion

In this study, we sequenced a draft genome of *C. clavata* BAUA-2787 and identified a 71 kb region as the KK-1 biosynthetic gene cluster, which consists of 10 ORFs including *kk1B*. The *kk1B* gene encodes an NRPS with a modular structure that is consistent with the structural features of KK-1. Furthermore, for the first time, we reported on the development of a transformation system for *C. clavata* using the AbA resistant gene *aurA* ^r^ that is carried on the pAUR316 plasmid as a drug-resistant marker or the homologous *pyrG* gene on *C. clavata* BAUA-2787 as an auxotrophic marker. It was also shown that it is possible to modify the target region of the genome by homologous recombination. Consequently, the transformation systems that were developed in this work were used to confirm experimentally that six of the 10 ORFs are essential for KK-1 biosynthesis and the others play important roles in its enhancement.

In general, especially for bacterial NRPSs, the TE domain that is present at the C-terminus in the final module of NRPS is known to catalyze hydrolysis or macrocyclization to release the peptide (Hur et al., 2012; Duban et al., 2022) but the C-terminus of the NRPS that is encoded by *kk1B* lacks a TE domain and instead contains a C domain. It has been revealed that many of the NRPSs that are found in fungal genomes contain a condensation-like domain (C_T_ domain) instead of a TE domain at the C-terminus, and that this domain is responsible for peptide macrocyclization (Gao et al., 2012). From these reports, we can speculate that in the NRPS that is encoded by *kk1B*, its C domain at the C-terminus in the final module is also responsible for releasing the peptide chain through macrocyclization.

The protein encoded by *kk1F* was predicted to have a bZIP domain and was the only gene encoding a transcription factor that was found within the putative KK-1 biosynthetic gene cluster. It is known that secondary metabolite clusters often contain a pathway specific transcription factor, which commonly regulate the expression of other cluster genes (Keller, 2019). A Zn(II)_2_Cys_6_-type transcription factor is well known as a pathway-specific transcription factor in fungi, such as *AflR* in the biosynthetic cluster of aflatoxin and its precursor sterigmatocystin in *Aspergillus* species (Yu et al., 1996; Flaherty and Payne, 1997; Ehrlich et al., 1999). Nevertheless, there have also been reported cases involving bZip-type transcription factors (Wang et al., 2008; Gao et al., 2011), suggesting that the protein encoded by *kk1F* likely regulates the expression of other genes in the KK-1 cluster. Therefore, *kk1F* overexpression and deletion strains were generated in this study, and we attempted to analyze the function of *kk1F* using these mutants. The results showed that the expression of the nine genes, except for *kk1F*, which constitutes the putative KK-1 cluster, was cooperatively regulated by the protein encoded by *kk1F* (**Figures 7B**, **8B**), strongly supporting that these are the biosynthetic cluster genes of KK-1. Additionally, *orf(-2)* and *orf(+10)*, located in the outer neighborhood of the cluster, also appeared to be co-expressed with the above nine genes. The protein encoded by *orf(-2)* shows similarity to the uncharacterized proteins of several plant pathogens, and that which is encoded by *orf(+10)* shows high similarity to known GDP-mannose transporters (**Table 1**). Although their involvement in KK-1 biosynthesis is not clear from this information alone and it has not been experimentally confirmed in this report, these two genes that are located on both ends of the KK-1 cluster may be strictly involved in KK-1 biosynthesis.

The four ORFs that were found in the KK-1 cluster, *kk1A*, *kk1D*, *kk1H*, and *kk1I*, probably contain the genes that are functionally required for KK-1 structure formation, because the KK-1 productivity of each deletion mutant was significantly reduced when compared to that of the wild-type strain (**Figure 9**). In particular, since three of the constituent residues of KK-1, D-lactic acid, L-pipecolic acid, and O-methyl-L-tyrosine, could not be synthesized by the enzyme domain of the NRPS encoded by *kk1B* alone, it was presumed that some of the above four genes were responsible for the biosynthesis of these residues.

**Figure 11:**
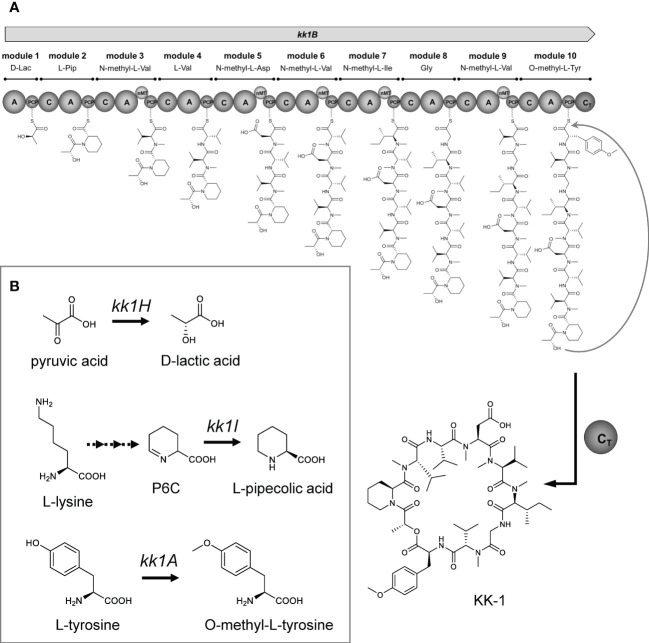
The proposed biosynthetic pathway for KK-1. **(A)** The proposed model for the KK-1 non-ribosomal peptide synthetase assembly line: C, condensation domain; A, adenylation domain; PCP, peptidyl carrier protein domain; nMT, nitrogen methyl transferase; CT, condensation-like domain. **(B)** The proposed pathway for the biosynthesis of non-amino acids or non-proteinogenic amino acids building blocks.

In terms of the NRPSs that synthesize cyclic depsipeptides, there are known cases in which a certain A domain recognizes and incorporates an α-hydroxy carboxylic acid that has been previously synthesized by some enzyme instead of an amino acid. In this case, a cyclic depsipeptide, with an ester bond, is released through the cyclization of the peptide chain with the hydroxy group at the α-position of this residue as a nucleophile in the terminal module of the NRPS. For the biosynthesis of beauvericin, a cyclic depsipeptide that is produced by the entomopathogenic fungus *Beauveria bassana*, the A domain of the first module of NRPS that is encoded by the *bbBeas* gene was presumed to recognize and activate D-α-hydroxyisovaleric acid. This D-α-hydroxyisovaleric acid is synthesized from valine catabolism or pyruvate metabolism by NADPH-dependent α-ketoisovalerate reductase, which is encoded by the *kivr* gene, which is located upstream and adjacent to *bbBeas* (Xu et al., 2008). This has been experimentally confirmed using the *kivr* knockout strain (Xu et al., 2008). The cyclic depsipeptide antibiotic valinomycin, which is produced by *Streptomyces tsusimaensis* ATCC 15141 contains D-α-hydroxyisovaleric acid and L-lactic acid residues, both of which are presumably activated by the A domain within the corresponding module of the NRPSs that are encoded by *vlm1* and *vlm2* (Cheng, 2006). The synthesis of these residues is probably not derived from stand-alone enzymes but it is speculated to involve unusual enzyme domains that occur in the NRPSs (Cheng, 2006). The protein encoded by *kk1H* in the KK-1 cluster contains the catalytic domain of a D-isomer specific 2-hydroxyacid dehydrogenase and has high homology to known D-lactate dehydrogenase (**Table 1**). This enzyme functions to catalyze the reversible conversion of pyruvate into lactic acid, and it is likely to be involved in the synthesis of D-lactic acid, a constituent residue of KK-1 (**Figure 11B**). During the biosynthesis of KK-1, it is considered that the A domain (in the first module of NRPS that is encoded by *kk1B*) first activates free D-lactic acid, which is synthesized from pyruvic acid by the D-lactate dehydrogenase which is encoded by *kk1H*, and then it is incorporated into the PCP domain of the same module (**Figure 11A**). After a series of peptide elongation reactions to synthesize the precursor peptide chain, cyclization with the hydroxy group of the D-lactic acid as a nucleophile is presumed to occur in the final module of NRPS, resulting in the formation of the depsipeptide ester bond.

L-pipecolic acid, a constituent residue of KK-1, is biosynthesized from L-lysine, and several biosynthetic pathways have been identified in microorganisms (He, 2006). One is the pathway that directly biosynthesizes L-pipecolic acid from L-lysine by the catalysis of lysine cyclodeaminase. The proteins encoded by *rapL* found in the biosynthetic gene cluster of rapamycin (Gatto et al., 2006) and encoded by *fkbL* found in the biosynthetic gene cluster of tacrolimus (Motamedi and Shafiee, 1998) were known to be involved in the synthesis of pipecolic acid residues as a lysine cyclodeaminase. The following other pathways are also known: the spontaneous cyclization of aldehydes generated by the deamination of the α-position of lysine or the elimination of the ϵ-amino group *via* saccharopine as a metabolic intermediate in accordance with the lysine metabolic pathway, resulting in P2C (Δ^1^-piperideine-2-carboxylate) or P6C (Δ^1^-piperideine-6-carboxylate), respectively. These are further reduced by specific enzymes to generate L-pipecolic acid (He, 2006). For the P6C pathway, there are also known cases in which P6C is generated directly from lysine by ϵ-aminotransferase (He, 2006). It has been shown that the reduction of P6C to L-pipecolic acid in microorganisms is at least partially catalyzed by pyrroline-5-carboxylate reductase (Fujii et al., 2002), which is known to be universally present in almost all organisms and is responsible for the conversion of Δ^1^-pyrroline-5-carboxylate to L-proline as the final reaction in L-proline biosynthesis (Delauney and Verma, 1993; Csonka and Leisinger, 2007). For the cyclic tetrapeptide apicidin F, a known histone deacetylase inhibitor that is produced by *Fusarium fujikuroi*, the biosynthesis of its pipecolic acid residue has been experimentally confirmed to involve the *APF3* gene that encodes pyrroline-5-carboxylate reductase, which is found in the biosynthetic gene cluster (Niehaus et al., 2014). The protein encoded by *kk1I* in the KK-1 biosynthetic cluster showed high sequence homology to known pyrroline-5-carboxylate reductase (**Table 1**), suggesting that this is an important gene for the synthesis of the L-pipecolic acid residue of KK-1 (**Figure 11B**). Thus, it is presumed that P6C, which is generated as a metabolic intermediate of lysine, is converted to L-pipecolic acid by the catalysis of pyrroline-5-carboxylate reductase, which is encoded by *kk1I*, and then it is recognized and incorporated by the A domain of the second module of NRPS, which is encoded by *kk1B* (**Figure 11A**).

The *kk1A* gene was predicted to encode a S-adenosyl-L-methionine-dependent methyltransferase based on sequence information and to have a conserved domain of O-methyltransferase (**Table 1**). S-adenosyl-L-methionine-dependent methyltransferases can transfer a methyl group from S-adenosyl-L-methionine, a universal methyl group donor in cells, to a nucleophilic acceptor such as C, O, N, or S, and they are involved in methylation in the biosynthesis of secondary metabolites (Roje, 2006). For the biosynthesis of skyllamycin, a cyclic depsipeptide that is produced by *Streptomyces* sp. Acta 2897 and has an O-methyltyrosine residue, it has been shown that the biosynthetic cluster gene *sky37* (encoding S-adenosyl-L-methionine-dependent methyltransferase) is responsible for the O-methylation of tyrosine as a free amino acid before its activation as an aminoacyl-AMP by the corresponding A domain of NRPS (Pohle et al., 2011). Similarly, in KK-1, we hypothesized that *kk1A* is responsible for the synthesis of the O-methyl-L-tyrosine residues. To demonstrate this, the culture metabolites of the wild-type strain and each strain with cluster gene deletion were compared (**Figure 10**). The results showed that the culture extracts of each strain contained at least five metabolites (compound a, b, c, d, and e) that were probably mono-demethylated forms of KK-1, all of which were basically produced at less than 1/20th of the amount of KK-1. In contrast, for the *kk1A* deletion strain, only the production of compound c was unchanged from that of the wild-type strain, although the productivity of KK-1 was significantly reduced when compared to the wild-type strain. KK-1 contains six methylated amino acid residues: three N-methyl-L-Val and one of N-methyl-L-Asp, N-methyl-L-Ile, and O-methyl-L-Tyr each, of which the five N-methylated amino acids are synthesized by the function of the corresponding nMT domains of NRPS. If the mono-demethylated metabolites that were detected in the culture extracts of each *C. clavata* BAUA-2787 strain are by-products that were synthesized due to a low rate of recognition and incorporation of the unmethylated amino acids by each A domain, which should activate the methylated amino acids, compound c would be the demethylated form at the O-methyltyrosine residue of KK-1. In other words, while the deletion of *kk1A* prevented the conversion of free tyrosine to O-methyltyrosine and, consequently, the synthesis of KK-1, it did not affect the incorporation pattern of unmethylated normal tyrosine by the A domain of the 10th module of NRPS, which should correspond to the O-methyltyrosine residue. Thus, the productivity of compound c was presumably unchanged from that of the wild-type strain. These results strongly suggest that intracellular tyrosine is pre-converted to O-methyl-L-tyrosine by the function of O-methyltransferase, which is encoded by *kk1A* (**Figure 11B**), and is recognized and incorporated by the A domain of the 10th module of NRPS, which is encoded by *kk1B* (**Figure 11A**).

The four ORFs that were found in the KK-1 cluster, *kk1C*, *kk1E*, *kk1G*, and *kk1J*, were considered to be involved in KK-1 production in some way by promoting or assisting productivity because their KK-1 productivity was maintained at a certain level even though it was lower than that of the wild-type strain (**Figure 9**). The *kk1E* gene was a putative gene of 234 bp, which encoded a protein with a small molecular weight of 8.1 kDa and showed high homology to the N-terminal sequence of known GDP-mannose transporters (**Table 1**). The RNA-Seq data of each strain showed that although transcription at the *kk1E* locus was observed, the sequencing reads were only mapped to the region from 5’UTR to approximately 160 bp downstream of the start codon (data not shown), suggesting that its 3’ terminus was not transcribed at all. It is possible that *kk1E* is not a functional gene and that the transcript from this locus regulates the expression of some gene that is involved in the production of KK-1, such as non-coding RNAs. The protein encoded by *kk1G* is predicted to be an ATP-binding cassette (ABC) transporter (**Table 1**), comprising two transmembrane domains and two nucleotide-binding domains. Since this gene is regulated with other cluster genes by *kk1F* encoding the transcription factor, it probably functions as a KK-1-specific transporter and is responsible for the extracellular efflux of the produced KK-1. The function of this transporter was presumed to confer self-tolerance against KK-1 but *kk1G* deletion had no effect on the fungal growth (data not shown). It was also assumed that the accumulation of KK-1 in the cells due to a lack of the efflux system would cause the suppression of cluster gene expression but the final production of KK-1 in the *kk1G* deletion strain was only 20–30% lower than that in the wild-type strain (**Figure 9**). These findings suggest that the protein encoded by *kk1G* plays a role in promoting KK-1 efflux. This is similar to the function of the ABC transporter gene, *sirA*, in the biosynthetic gene cluster of the fungal toxin sirodesmin, which is produced by the plant pathogen *Leptosphaeria maculans* (Gardiner et al., 2005). It is also possible that there is another efflux system outside the KK-1 cluster, such as *atrD*, which encodes an ABC transporter that functions as a multiple drug resistance factor in *Aspergillu nidulans* (Andrade et al., 2000). The protein encoded by *kk1J* is predicted to belong to the α/β-hydrolase superfamily, and the Pfam search revealed that it contains the conserved domain of thioesterase (**Table 1**). Such thioesterases that are presented alone in the biosynthetic gene cluster of secondary metabolites are called type II thioesterases (TEIIs; Kotowska and Pawlik, 2014) and are distinguished from type I thioesterase, which is integrated into enzyme complexes such as NRPS and polyketide synthase (PKS) to release the synthesized products (Hur et al., 2012; Duban et al., 2022). Although, in most cases, TEIIs are not essential for the biosynthesis of the corresponding secondary metabolites, they are known to be important for efficient production because of their role in correcting products that are synthesized by NRPS and PKS (Schneider and Marahiel, 1998; Schwarzer et al., 2002; Kotowska and Pawlik, 2014). It is known that acyl residues that are incorrectly loaded into PCP domains block subsequent peptide chain elongation and cause the disruption of the module function of NRPS but TEII can remove them by hydrolysis to restore normal module function (Schwarzer et al., 2002; Yeh et al., 2004). Thus, in the biosynthesis of KK-1, TEII encoded by *kk1J* is likely to be involved in the corrective mechanism of peptide chain synthesis.

Currently, it is unclear how *kk1C* and *kk1D* are involved in the production of KK-1. In particular, the function of the protein encoded by *kk1D* is completely unknown because there is no similar protein with a known function but its role is very interesting because it is considered essential for KK-1 biosynthesis based on KK-1 productivity in the deletion strain (**Figure 9**).

Although further studies of the culture metabolites in each gene deletion or overexpression mutant are needed to more precisely understand the functions of the 10 ORFs that were found in the KK-1 cluster, it was revealed that they are at least involved in KK-1 production in some way. Yoshimi et al. (2018) succeeded in the heterologous production of KK-1 in *Aspergillus oryzae* in which eight of these 10 ORFs (except for *kk1F*, which encodes the specific transcription factor for the KK-1 pathway, and *kk1E*, which is probably not a gene) were all introduced under the control of the maltose-inducible *amyB* promoter. This provides further support that these genes are required for KK-1 biosynthesis. These results indicate that the efficient production of KK-1 may be achieved by studying the appropriate heterologous hosts and by using synthetic biology approaches to build metabolic pathways that are suitable for KK-1 production within those hosts. In this study, the *C. clavata* BAUA-2787 mutant in which *kk1F* was overexpressed under the control of the *Ccnmt1* promoter showed approximately twice as much KK-1 production as the wild-type strain after 7 days of cultivation. Therefore, this suggests that further optimization of the promoter to express *kk1F* could lead to a drastic improvement in KK-1 productivity. Thus, the successful identification of the KK-1 biosynthetic gene cluster has enabled diverse approaches for the future realization of the mass production of KK-1.

## Data availability statement

The datasets presented in this study can be found in online repositories. The names of the repository/repositories and accession number(s) can be found below: https://www.ncbi.nlm.nih.gov/genbank/, LC371755, https://www.ncbi.nlm.nih.gov/, BBC83956.1, https://www.ncbi.nlm.nih.gov/, BBC83957.1, https://www.ncbi.nlm.nih.gov/, BBC83958.1, https://www.ncbi.nlm.nih.gov/, BBC83959.1, https://www.ncbi.nlm.nih.gov/, BBC83960.1, https://www.ncbi.nlm.nih.gov/, BBC83961.1, https://www.ncbi.nlm.nih.gov/, BBC83962.1, https://www.ncbi.nlm.nih.gov/, BBC83963.1, https://www.ncbi.nlm.nih.gov/, BBC83964.1, https://www.ncbi.nlm.nih.gov/, BBC83965.1.

## Author contributions

SY, TF, KA, MM and KK conceived and designed the experiments. SY, TF and TK carried out the genome sequence of the fungus. SY and TF performed the construction of the fungal strains. SY performed the essential experiments and analyzed the data. MU analyzed the data. SY, AY, KA, and KK wrote the paper. All authors contributed to the article and approved the submitted version.
