# Supplementary material for: Discovery of a gene cluster for the biosynthesis of novel cyclic peptide compound, KK-1, in Curvularia clavata
Source: Front Fungal Biol. 2023 Jan 20;3:1081179. doi: 10.3389/ffunb.2022.1081179 (PMC10512319; doi:10.3389/ffunb.2022.1081179)
Supplement: Supplementary file 1 [file DataSheet_1.docx]

Supplementary Material

# Supplementary Figures


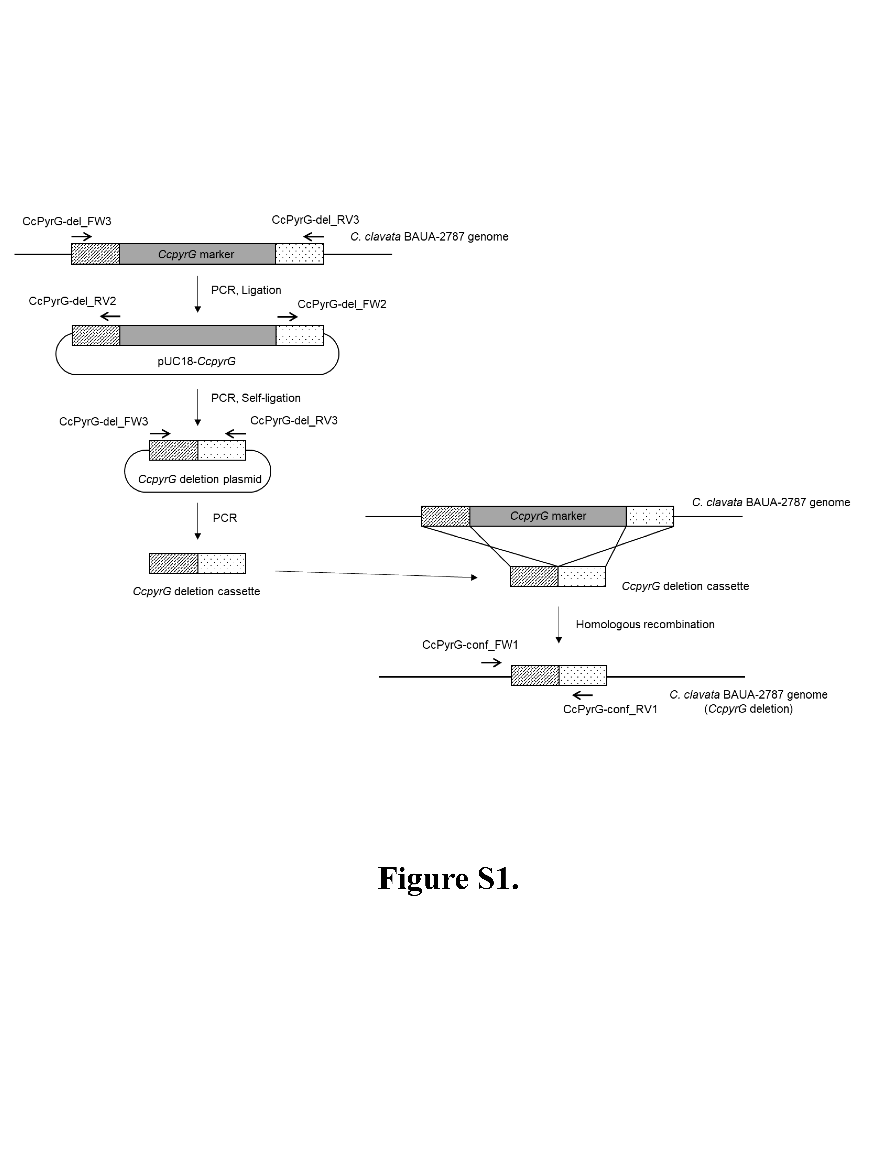


**Supplementary Figure 1.** Scheme of *CcpyrG* deletion by homologous recombination. "*CcpyrG* marker" refers to the region probably containing from the promoter to the terminator of *CcpyrG*. The arrows indicate the positions where the primers used for PCR to prepare the deletion cassette or to confirm that the desired strains have been obtained anneal.


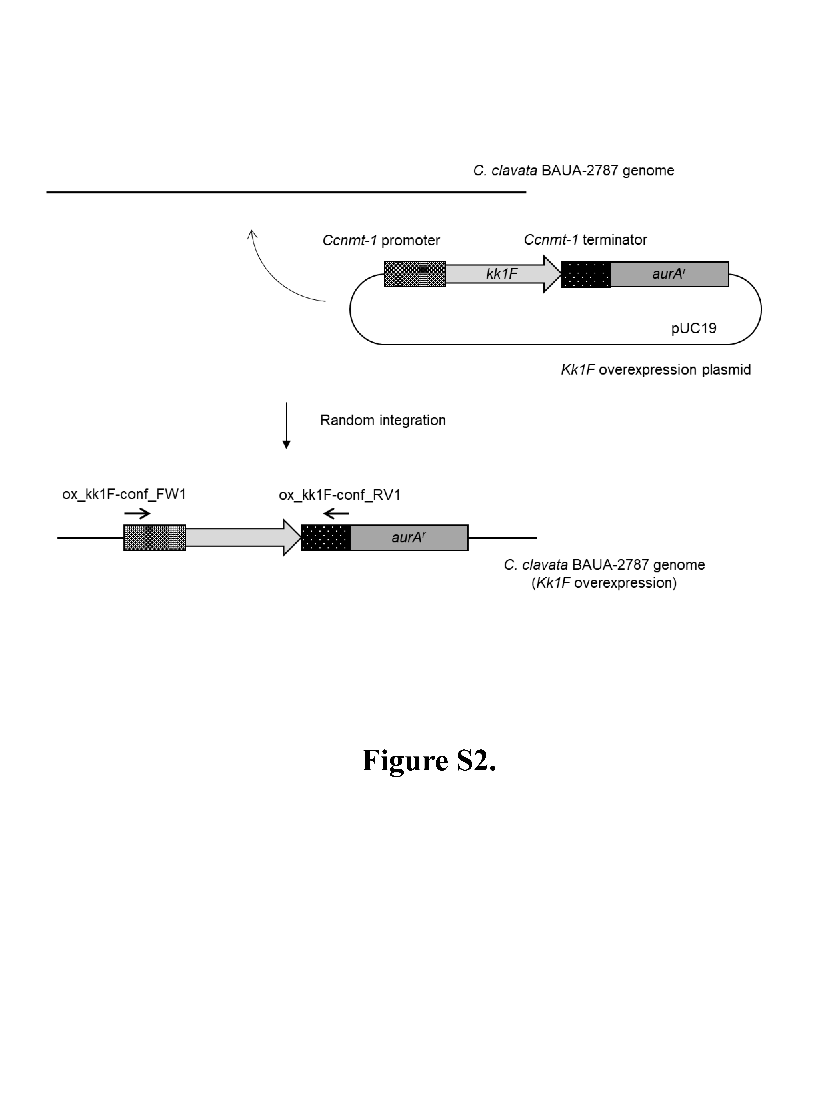


**Supplementary Figure 2.** Scheme of *kk1F* overexpression. The arrows indicate the positions where the primers used for PCR to confirm that the desired strains have been obtained anneal.


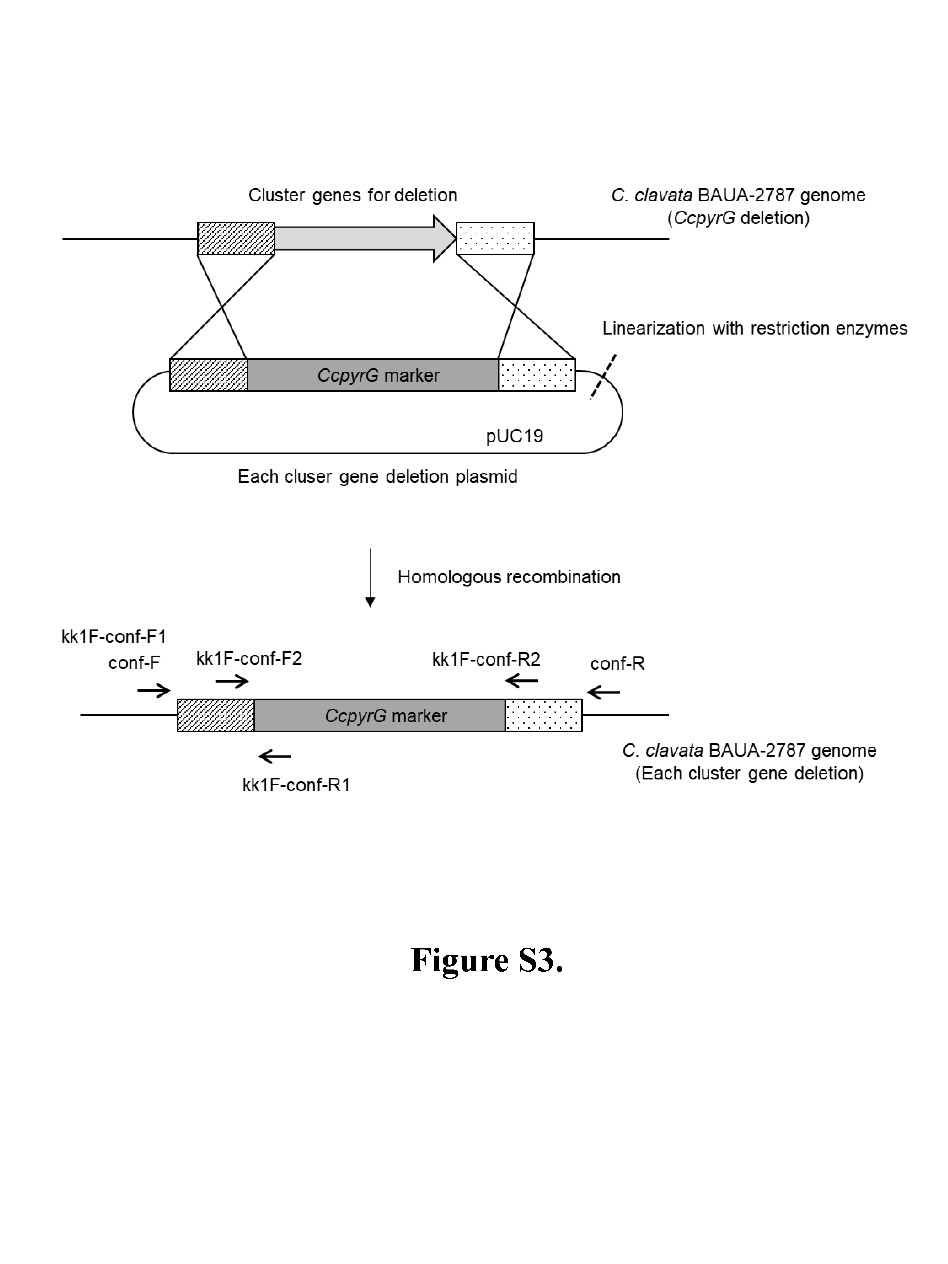


**Supplementary Figure 3.** Scheme of each cluster gene deletion except *kk1B* by homologous recombination. The arrows indicate the positions where the primers used for PCR to confirm that the desired strains have been obtained anneal.


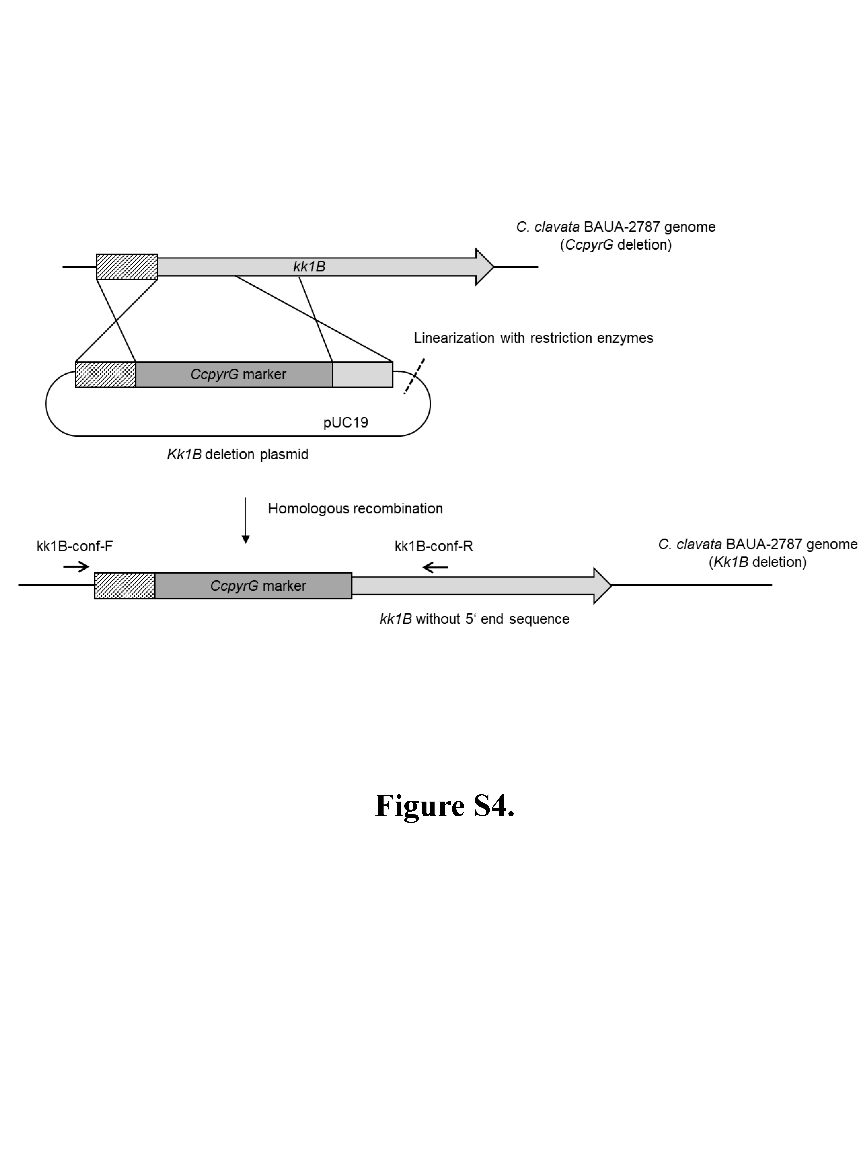


**Supplementary Figure 4.** Scheme of *kk1B* deletion by homologous recombination. The arrows indicate the positions where the primers used for PCR to confirm that the desired strains have been obtained anneal.


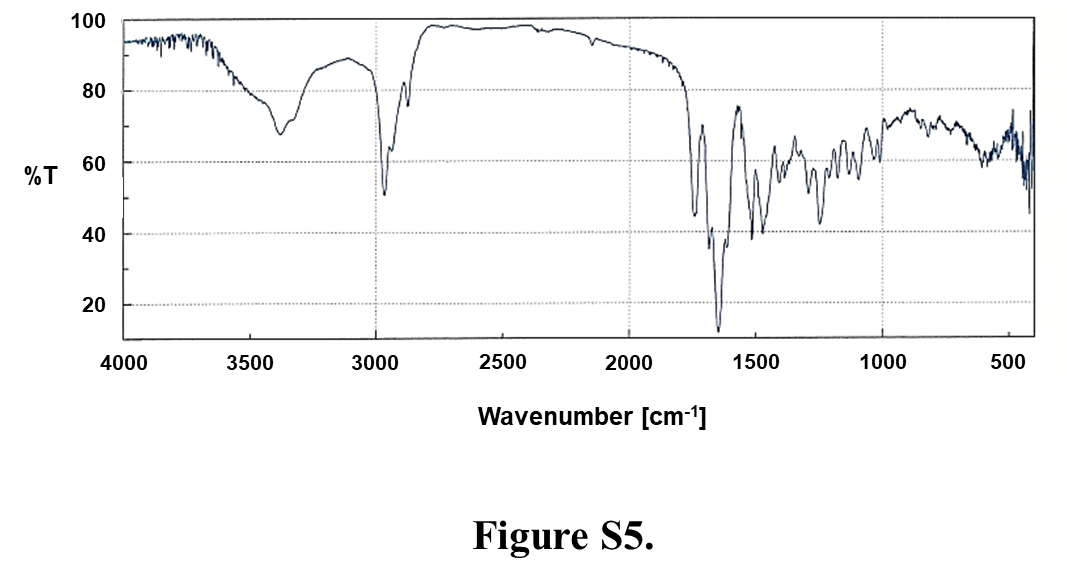


**Supplementary Figure 5.** Infrared (IR) spectrum of KK-1 recorded on a FT/IR-460 spectrometer (JASCO, Tokyo, Japan) using KBr pellets. The analytically pure KK-1 used here was obtained from the culture extract by silica gel column chromatography using hexane/ethyl acetate (4:1 to 1:3) as the eluent and by subsequent recrystallization from diisopropyl ether. This IR spectrum matches that of BK202 described in the patent WO1992005191A1, Fig. 6.


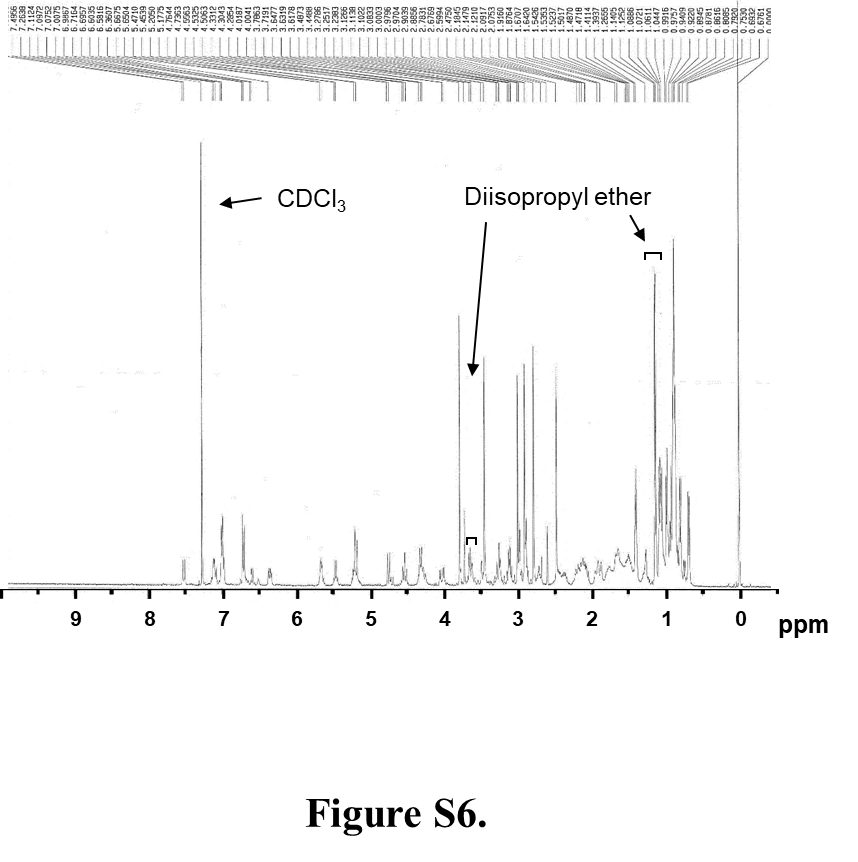


**Supplementary Figure 6.** Proton nuclear magnetic resonance (^1^H-NMR) spectrum of KK-1 in CDCl_3_ recorded at 400 MHz on a JNM-LA 400 spectrometer (JEOL, Tokyo, Japan). The analytically pure KK-1 used here was obtained from the culture extract by silica gel column chromatography using hexane/ethyl acetate (4:1 to 1:3) as the eluent and by subsequent recrystallization from diisopropyl ether. The peaks derived from CDCl_3_ and diisopropyl ether are indicated in the figure. This NMR spectrum matches that of BK202 described in the patent WO1992005191A1, Fig. 2.

# Supplementary Tables

**Table S1. Primers used in this study**

| Name | Sequence (5’-3’)  *15 bp overlap regions for In-Fusion cloning are underlined. |
| --- | --- |
| **Confirmation of pUAR316 introduction** | |
| AnaurA_FW1 | GGAGTATGGGTATGGTGCAG |
| AnaurA_RV1 | CAACGGCGTGTAGCGATGAG |
| **Construction of the DNA fragment for *CcpyrG* deletion** | |
| CcPyrG-del_FW3 | GACAGACTCTTCGTCGACGTC |
| CcPyrG-del_RV3 | GTTGTGGTTGGTGTTCCTGAGG |
| CcPyrG-del_FW2 | CACTCGATCTACCAAATCGACG |
| CcPyrG-del_RV2 | CCTATCCGGATATGCAGTCAC |
| **Confirmation of the *CcpyrG* deletion strain** | |
| CcPyrG-conf_FW1 | GGCTTCTTGGACTTCTTCTTGGG |
| CcPyrG-conf_RV1 | AAGTGACGACCGGAGACAGTAGG |
| **Construction of the plasmid for *kk1F* overexpression** | |
| nmt1-pro_In-Fus_FW1 | CGGTACCCGGGGATCTAGTCTGTTGATTACTCG |
| nmt1-pro_In-Fus_RV1 | CTCGACAAAGGTCATTTTGACTTTGAATACCGGTG |
| nmt1-ter_FW1 | GCAGTTGCCGTTGGACCAGAGG |
| nmt1-ter_In-Fus | ATAGTCATAACAAGCCGCGACACTGTAATATTAAAGC |
| TF-CDS_FW1 | ATGACCTTTGTCGAGACTGTAGCC |
| TF-CDS_In-Fus_RV1 | TCCAACGGCAACTGCCTATGATATACTCATGTTCTCGTC |
| AnaurA-mark_In-Fus_FW1 | CGACTCTAGAGGATCCTGATGGTCAGATGGATCTG |
| AnaurA-mark_RV1 | GCTTGTTATGACTATGTATACATATGCG |
| **Confirmation of the *kk1F* overexpression strain** | |
| ox_kk1F-conf_FW1 | TAGTCTGTTGATTACTCG |
| ox_kk1F-conf_RV1 | CGCGACACTGTAATATTAAAGC |
| **Construction of the plasmid for each cluster gene deletion** | |
| CcPyrG-mark_FW1 | CATGACGGTTGCTAGGGTCG |
| CcPyrG-mark_RV1 | GCCGCTCTGCTTCATTGCTG |
| kk1A_del_L-arm_FW | CGGTACCCGGGGATCGACCCATTGCAGCTTGTG |
| kk1A_del_L-arm_RV | ATGAAGCAGAGCGGCGTGCAGTATGGTGTCTAAAACG |
| kk1A_del_R-arm_FW | CTAGCAACCGTCATGGATGAATGAGCACCCTGTTAG |
| kk1A_del_R-arm_RV | CGACTCTAGAGGATCGTACATTACAAAAACCTGTTGCAG |
| kk1B_del_L-arm_FW | CGGTACCCGGGGATCGTCCCACGTGCAGCTTCAAC |
| kk1B_del_L-arm_RV | ATGAAGCAGAGCGGCCGTGGAGTATCCCAGGATGG |
| kk1B_del_R-arm_FW | CTAGCAACCGTCATGCCAGCCAAAGGGTATCATGG |
| kk1B_del_R-arm_RV | CGACTCTAGAGGATCTGAGGGCAGCGTAGCCTG |
| kk1C_del_L-arm_FW | CGGTACCCGGGGATCGTGGATAAATTCGTACCCTTTG |
| kk1C_del_L-arm_RV | ATGAAGCAGAGCGGCCTGATCTTTGTTGTGGTCGTG |
| kk1C_del_R-arm_FW | CTAGCAACCGTCATGCAGTTTGGCACTTGAGCATC |
| kk1C_del_R-arm_RV | CGACTCTAGAGGATCCACGGAAAGGAACTCCTACAG |
| kk1D_del_L-arm_FW | CGGTACCCGGGGATCCTCTGGGAAAAGCGGTTAG |
| kk1D_del_L-arm_RV | ATGAAGCAGAGCGGCGAAGAACCGAGAGCGAGAG |
| kk1D_del_R-arm_FW | CTAGCAACCGTCATGCTTGCATCTACCTAGATATTTCACG |
| kk1D_del_R-arm_RV | CGACTCTAGAGGATCCAGAGAATCAGCAGAGACACC |
| kk1E_del_L-arm_FW | CGGTACCCGGGGATCCCCTGGTAGTTCAGTGGAAGTAAG |
| kk1E_del_L-arm_RV | ATGAAGCAGAGCGGCTGATAGAGGTACGGGGGTG |
| kk1E_del_R-arm_FW | CTAGCAACCGTCATGTGCTTGGCTGCTTCAAATC |
| kk1E_del_R-arm_RV | CGACTCTAGAGGATCCTAATACTTGTCGTCCCACTGATG |
| kk1F_del_L-arm_FW | CGGTACCCGGGGATCCTCTGAAGCGGTCAAGGATAACG |
| kk1F_del_L-arm_RV | ATGAAGCAGAGCGGCGAGCCTAAGATATGCCAGGAGG |
| kk1F_del_R-arm_FW | CTAGCAACCGTCATGCCATAGACGTGGCACTCGAACG |
| kk1F_del_R-arm_RV | CGACTCTAGAGGATCCGTCTTAAGGATGGTTCAGCTGC |
| kk1G_del_L-arm_FW | CGGTACCCGGGGATCGCAGTACATCGTCAGGGTC |
| kk1G_del_L-arm_RV | ATGAAGCAGAGCGGCGATGAATAAGGCGAAGGAAAG |
| kk1G_del_R-arm_FW | CTAGCAACCGTCATGCCCTCTTTTTTCTTGCTGTCTC |
| kk1G_del_R-arm_RV | CGACTCTAGAGGATCGAAGGAAGGACGGATACTGG |
| kk1H_del_L-arm_FW | CGGTACCCGGGGATCGATGAGCGTAGAATTCGTAAAAAG |
| kk1H_del_L-arm_RV | ATGAAGCAGAGCGGCGCGAACGGGCGTTTTTC |
| kk1H_del_R-arm_FW | CTAGCAACCGTCATGGAAGGAAGGACGGATACTGG |
| kk1H_del_R-arm_RV | CGACTCTAGAGGATCCCCTCTTTTTTCTTGCTGTCTC |
| kk1I_del_L-arm_FW | CGGTACCCGGGGATCCTCCTTATTTTGCAACTTCTGATAC |
| kk1I_del_L-arm_RV | ATGAAGCAGAGCGGCCGTGTTGATTTTGGTAATTTTG |
| kk1I_del_R-arm_FW | CTAGCAACCGTCATGGATGAGCGTAGAATTCGTAAAAAG |
| kk1I_del_R-arm_RV | CGACTCTAGAGGATCGCGAACGGGCGTTTTTC |
| kk1J_del_L-arm_FW | CGGTACCCGGGGATCCGTGTTGATTTTGGTAATTTTG |
| kk1J_del_L-arm_RV | ATGAAGCAGAGCGGCCTCCTTATTTTGCAACTTCTGATAC |
| kk1J_del_R-arm_FW | CTAGCAACCGTCATGCTAGCAGCCATAAGAGACGTAACC |
| kk1J_del_R-arm_RV | CGACTCTAGAGGATCGTTTTCATTGCATGCTCCG |
| **Confirmation of each cluster gene deletion strain** | |
| kk1A-conf-F | AAGATAAGCACTGGTCCTGC |
| kk1A-conf-R | AACAGGACTAGGTAAGCTGC |
| kk1B-conf-F | CATTTACAGAAGGTGAGGC |
| kk1B-conf-R | AGAAGTTATCAGTGACGCC |
| kk1C-conf-F | GTAAACTTGTCGTTGGCGAG |
| kk1C-conf-R | CTTCACGTACTATGCAACCG |
| kk1D-conf-F | TTTCTGATCCTTGACGCC |
| kk1D-conf-R | TCGAGGATTTACCTACTGGG |
| kk1E-conf-F | GGAAGATTACATTCCCGTCC |
| kk1E-conf-R | ACTTATCCTAACTCCGCCCG |
| kk1F-conf-F1 | CACTGAACTACCAGGGGTTG |
| kk1F-conf-R1 | GAAAGCGCAGCAATGAAG |
| kk1F-conf-F2 | CTCCTGGCATATCTTAGGCTC |
| kk1F-conf-R2 | CGTATATCTGTGCGTTCGAG |
| kk1G-conf-F | CTACAAAGACCATTCGGG |
| kk1G-conf-R | TACAACCATAGACGTGGC |
| kk1H-conf-F | TGTAATCCGAGCTATGCC |
| kk1H-conf-R | ATCTGCAAATCCTACCGG |
| kk1I-conf-F | GTTGAAAGTTGGGATCTGGG |
| kk1I-conf-R | ATTCGAATTCCTGCCCTG |
| kk1J-conf-F | AATGCTAACCTCCAACGC |
| kk1J-conf-R | GTAATCCTCACCATTCCC |

**Table S2. Genomic regions used as the homology arms in each deletion plasmid and restriction enzymes used for their linearization**

|  | **Left arm (before start codon)** | | |  | **Right arm (behind stop codon)** | | |  | **Right arm (from start codon)** | | |  |  |
| --- | --- | --- | --- | --- | --- | --- | --- | --- | --- | --- | --- | --- | --- |
| **Target gene for deletion** | **from** | **to** | **length  (bp)** |  | **from** | **to** | **length (bp)** |  | **from** | **to** | **length (bp)** |  | **Restriction enzymes  for linearization** |
| *kk1A* | -983 | -2 | 982 |  | 6 | 955 | 950 |  |  |  |  |  | HindIII |
| *kk1B* | -1,012 | -13 | 1,000 |  |  |  |  |  | 4,482 | 5,463 | 982 |  | EcoRI |
| *kk1C* | -997 | -6 | 992 |  | 4 | 996 | 993 |  |  |  |  |  | EcoRI |
| *kk1D* | -912 | -1 | 912 |  | 1 | 995 | 995 |  |  |  |  |  | EcoRI |
| *kk1E* | -999 | -9 | 991 |  | 11 | 1,013 | 1,003 |  |  |  |  |  | EcoRI |
| *kk1F* | -1,235 | -89 | 1,147 |  | 59 | 1,263 | 1,205 |  |  |  |  |  | EcoRI |
| *kk1G* | -997 | -5 | 993 |  | 6 | 584 | 579 |  |  |  |  |  | KpnI |
| *kk1H* | -774 | -6 | 769 |  | 3 | 581 | 579 |  |  |  |  |  | HindIII |
| *kk1I* | -716 | -1 | 716 |  | 1 | 769 | 769 |  |  |  |  |  | HindIII |
| *kk1J* | -717 | -2 | 716 |  | 2 | 989 | 988 |  |  |  |  |  | EcoRI |
